# Spatial transcriptomics for profiling the tropism of viral vectors in tissues

**DOI:** 10.1038/s41587-022-01648-w

**Published:** 2023-01-26

**Authors:** Min J. Jang, Gerard M. Coughlin, Cameron R. Jackson, Xinhong Chen, Miguel R. Chuapoco, Julia L. Vendemiatti, Alexander Z. Wang, Viviana Gradinaru

**Affiliations:** 1 Division of Biology and Biological Engineering, California Institute of Technology, Pasadena, CA, USA.; 2 Division of Engineering and Applied Science, California Institute of Technology, Pasadena, CA, USA.

## Abstract

A barrier to advancing engineered adeno-associated viral vectors (AAVs) for precision access to cell subtypes is a lack of high-throughput, high-resolution assays to characterize in vivo transduction profiles. In this study, we developed an ultrasensitive, sequential fluorescence in situ hybridization (USeqFISH) method for spatial transcriptomic profiling of endogenous and viral RNA with a short barcode in intact tissue volumes by integrating hydrogel-based tissue clearing, enhanced signal amplification and multiplexing using sequential labeling. Using USeqFISH, we investigated the transduction and cell subtype tropisms across mouse brain regions of six systemic AAVs, including AAV-PHP.AX, a new variant that transduces robustly and efficiently across neurons and astrocytes. Here we reveal distinct cell subtype biases of each AAV variant, including a bias of AAV-PHP.N toward excitatory neurons. USeqFISH also enables profiling of pooled regulatory cargos, as we show for a 13-variant pool of microRNA target sites in AAV genomes. Lastly, we demonstrate potential applications of USeqFISH for in situ AAV profiling and multimodal single-cell analysis in non-human primates.

Targeted delivery of transgenes, encoding fluorescent reporters, functional sensors/effectors, supplemental proteins to rescue imbalanced cell functions or genome editors, to specific cell populations enables us to untangle complex biological circuits and offers promise as a therapeutic approach^1–4^. Targeting strategies include transgenic animal models, engineered viral vectors with biased tropisms^5–13^, gene regulatory elements (for example, promoters^14–18^, enhancers^19–25^ and/or microRNA target sites^26–28^) and various delivery routes. These methods, often used in combination^2,27^, have granted selective access to a few cell types but are still far from covering most molecularly distinct cell populations. Furthermore, specificity of gene delivery is crucial for therapeutic applications where off-target effects can be lethal. Therefore, there is a pressing need for an expanded toolkit for efficient, precisely targeted gene delivery.

Adeno-associated viruses (AAVs) can be efficient and specific vectors for in vivo gene delivery. Natural AAVs have been widely used in both research and clinical fields owing to their minimal pathogenicity, long-term persistence and serotypes with diverse infectivity profiles. Engineering AAV capsids through rational design and/or directed evolution has yielded useful variants for intravenous gene transfer efficiently to the central and peripheral nervous systems (for example, AAV-PHP.B^5^, AAV-PHP.eB^6^, AAV-F^11^ and TRACER capsids^12^ for the central nervous system (CNS) and AAV-PHP.S^6^ for the peripheral nervous system (PNS)) or preferentially to certain cell types (for example, AAV-PHP. N^7^ and AAV-CAP.B10^8^ for neurons; AAVMYO^13^ and MyoAAV^9^ for muscle; and AAV-PHP.V1^7^ for vascular endothelial cells) of rodents. Recently developed AAV vectors for efficient, minimally invasive genetic access to the non-human primate (NHP) brain (for example, AAV.CAP-B22 (ref. ^8^) for marmosets and AAV.CAP-Mac^29^ for rhesus macaques) or muscle (for example, MyoAAV^9^ for cynomolgus macaques) show the suitability of engineered AAVs for targeted gene delivery and their promise for clinical translation.

To this end, efforts to accelerate AAV engineering have advanced screening platforms^5,9,12,30^ (for example, M-CREATE^7^) and enabled high-throughput discovery of many promising variants; however, the subsequent tropism characterization remains low throughput and low resolution. Bulk assays with extracted DNA/RNA (for example, qPCR^5^ and next-generation sequencing^7–9,12,13,29,31^) can effectively narrow a library of capsid variants down to dozens of promising candidates and identify those with organ-specific tropism^8,13^, yet they lack single-cell resolution. Cell type tropism characterization has largely been restricted to immunohistochemistry (IHC) of a few major cell types due to limited cell type marker antibodies and fluorescence multiplexing. To address these limitations, we recently reported a method based on single-cell RNA sequencing (scRNA-seq) that enables deep characterization of pooled AAV variants in mouse cortex^32^, with the caveats that spatial patterns of both endogenous and viral gene expression are lost.

To enable high-throughput, high-resolution profiling of AAVs in intact tissue, we envisioned a spatial transcriptomic approach. Spatial transcriptomics is an emerging technology that enables readout of tens to thousands of endogenous nucleic acid sequences at single-molecule resolution in tissue sections while preserving spatial context^33–45^. Its utility has recently been extended to read out barcoded transgenes, enabling screening of genetic variant libraries in vitro^46,47^, mapping cell projections^48^ and tracing cell lineages over time^49,50^. Although in situ detection of AAV genomes after single/direct injection^51,52^ supports the feasibility of this approach for AAV profiling, multiplexed detection and quantitative analysis of barcoded systemic AAVs remain challenging; the multiplicity of infection (MOI) of each variant is low due to safety considerations that limit the total dose of a systemically administered pool. With the abundance of systemic AAV transcripts (10–100 transcripts per cell^32^) similar to that of endogenous genes, current methodologies for spatial transcriptomics require dozens of probes per target for sufficient specificity, necessitating a prohibitively long barcode (~0.5–1 kilobase (kb)) for the AAVs with a small packaging capacity (<4.7 kb).

To tackle these challenges, we developed a highly sensitive spatial transcriptomic method, called ultrasensitive sequential fluorescence in situ hybridization (USeqFISH; Fig. 1a). This method combines signal amplification and sequential labeling strategies to yield much brighter and more sensitive RNA signal than conventional amplification (for example, rolling-circle amplification (RCA)^53^ or hybridization chain reaction (HCR)^54^) with four, or even fewer, oligonucleotide probes per target gene, enabling short barcoding of AAV genomes and allowing readout of ~50 genes, sufficient to encompass multiple AAVs and endogenous genes to infer cell types in various brain regions^43,44,48^.

To demonstrate the ability of USeqFISH to profile systemic AAVs, we analyzed a pool of six variants with a range of efficiency and specificity in the mouse brain—the previously described AAV-PHP.eB^6^, AAV. CAP-B10 (ref. ^8^), AAV-PHP.N^7^, AAV-PHP.V1 (ref. ^7^) and AAV-PHP.B8 (ref. ^7^), as well as a variant, AAV-PHP.Across (abbreviated to AAV-PHP.AX), that has a tropism-refining peptide identified from phage display screens^55^ substituted in the AA452–485 loop of AAV-PHP.eB. USeqFISH recapitulated known characteristics of each variant, such as overall transduction efficiency and major cell type tropism, previously accrued over multiple studies, in a single experiment. USeqFISH also revealed relatively distinct cell subtype tropisms of each variant across multiple brain regions (cortex, striatum, thalamus and cerebellum). Notably, we found that AAV-PHP.AX efficiently and broadly transduces neuronal subtypes and astrocytes, with higher flexibility for tuning tropism when paired with gene regulatory elements. We also expand the applicability of USeqFISH into the in situ profiling of pooled regulatory cargos in tissue by demonstrating the distinct regulation effect of microRNA target sites (miRNA TSs) inserted in the AAV genome across cell types as an example. Finally, we demonstrate the utility of USeqFISH in the NHP brain with viral tools, illustrating the generalizability of USeqFISH for various applications, such as in situ screening of AAV capsid and cargo variant pools and integrative analysis of cellular barcoding/typing/tracing in intact tissue across species.

## Results

### USeqFISH for in situ profiling of endogenous and viral genes

**Fig. 1 |:**
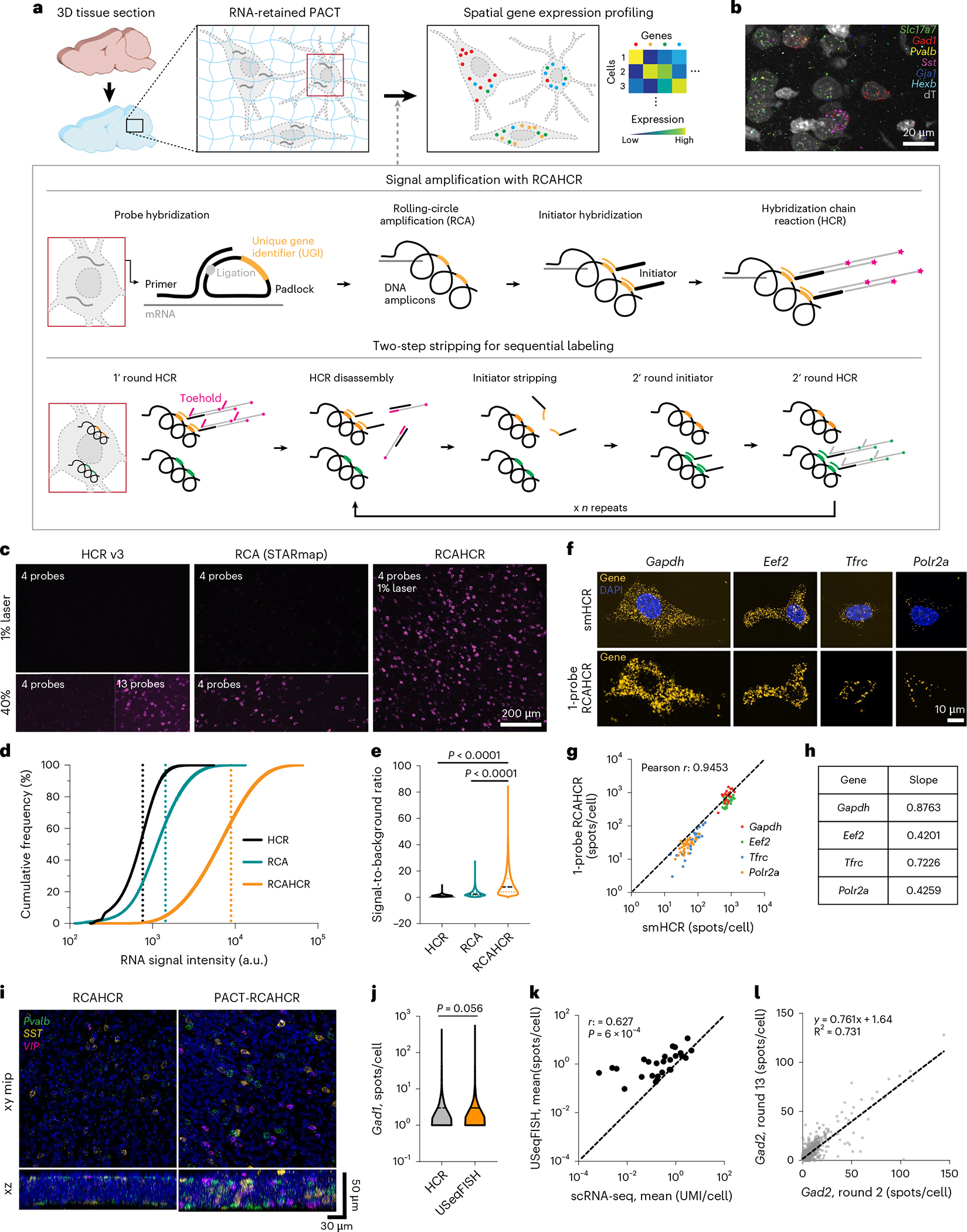
USeqFISH for highly sensitive, spatial gene expression profiling in 3D, intact tissue. **a**, Schematic procedure of USeqFISH. **b**, Representative maximum intensity projection image of USeqFISH with six selected genes and a cytosolic marker (dT) in mouse cortex. **c**, In situ detection of *Gad1* with four probes using HCR version 3, RCA (STARmap) and RCAHCR amplification. The same region was imaged twice with 1% and 40% laser power. For HCR, we confirmed its ability to detect RNA by adding more probes (13 probes total). **d**, Cumulative distribution of RNA signal intensity of HCR-only, RCA-only and RCAHCR amplification. Dashed lines indicate the mean of each distribution (a.u., arbitrary units). **e**, SBR of HCR-only, RCA-only and RCAHCR amplification (two-sided unpaired *t*-test with Welch’s correction). **f**, Comparison of smHCR and subsequent 1-probe RCAHCR with four housekeeping genes (*Gapdh*, *Eef2*, *Tfrc* and *Polr2a*) in the same NIH3T3 cells. **g**, The quantification of spots per cell detected by smHCR and subsequent 1-probe RCAHCR. The Pearson correlation coefficient (*r*) of four genes was 0.9453. **h**, The slope of each gene in **g**. **i**, Comparison of RCAHCR penetration without (‘RCAHCR’) and with RNA-retained PACT clearing (‘PACT-RCAHCR’) in 50-μm-thick brain tissue. We labeled three genes (*Pvalb*: green, *Sst*: yellow and *Vip*: magenta) with three orthogonal hairpin pairs in the same tissue. **j**, Comparison of *Gad1* spot numbers per cell detected with HCR (gray; *n* = 2,322 cells) and with USeqFISH (orange; *n* = 3,403 cells; two-sided unpaired *t*-test with Welch’s correction). **k**, Comparison of the expression level of 26 endogenous genes (Supplementary Table 1) measured as mean spot numbers per cell with USeqFISH and mean UMI counts per cell with scRNA-seq (*r*: Pearson correlation coefficient; *P*: *P* value). The dashed line indicates *x* = *y*. **l**, Comparison of *Gad2* spot numbers per cell detected with USeqFISH at round 2 and round 13 in the same tissue. Each spot indicates the same cell. The dashed line indicates linear regression.

To enable spatial transcriptomic approaches for high-throughput, high-resolution AAV tropism profiling, we developed USeqFISH (Fig. 1a,b). USeqFISH comprises a signal amplification step with combined RCA^45^ and HCR^54^ (RCAHCR; Fig. 1a, ‘Signal amplification with RCAHCR’) and a sequential labeling step with a two-step hairpin and initiator stripping method via toehold-mediated strand displacement (Fig. 1a, ‘Two-step stripping for sequential labeling’).

The new amplification strategy, RCAHCR, combines two conventional signal amplification methods (RCA and HCR) to achieve high sensitivity. Adapting the SNAIL probe of STARmap^40^, we designed four probes for each gene, with each probe consisting of a primer and a padlock. Each primer and padlock include 20 nucleotides (nt) complementary to consecutive sequences of the target. This paired design offers higher specificity, as the RCA amplification can happen only if both primer and padlock have hybridized on the target^40,54^. The padlock also has a 19-nt unique gene identifier (UGI) that is replicated via RCA and to which the initiator (an HCR initiator with sequence complementary to the UGI, UGI*) hybridizes. RCAHCR is, therefore, carried out by hybridizing probes to the target gene, generating DNA amplicons via RCA, hybridizing the initiators to the amplicons and, lastly, triggering spontaneous HCR assembly by adding hairpins.

**Extended Data Fig. 1 |:**
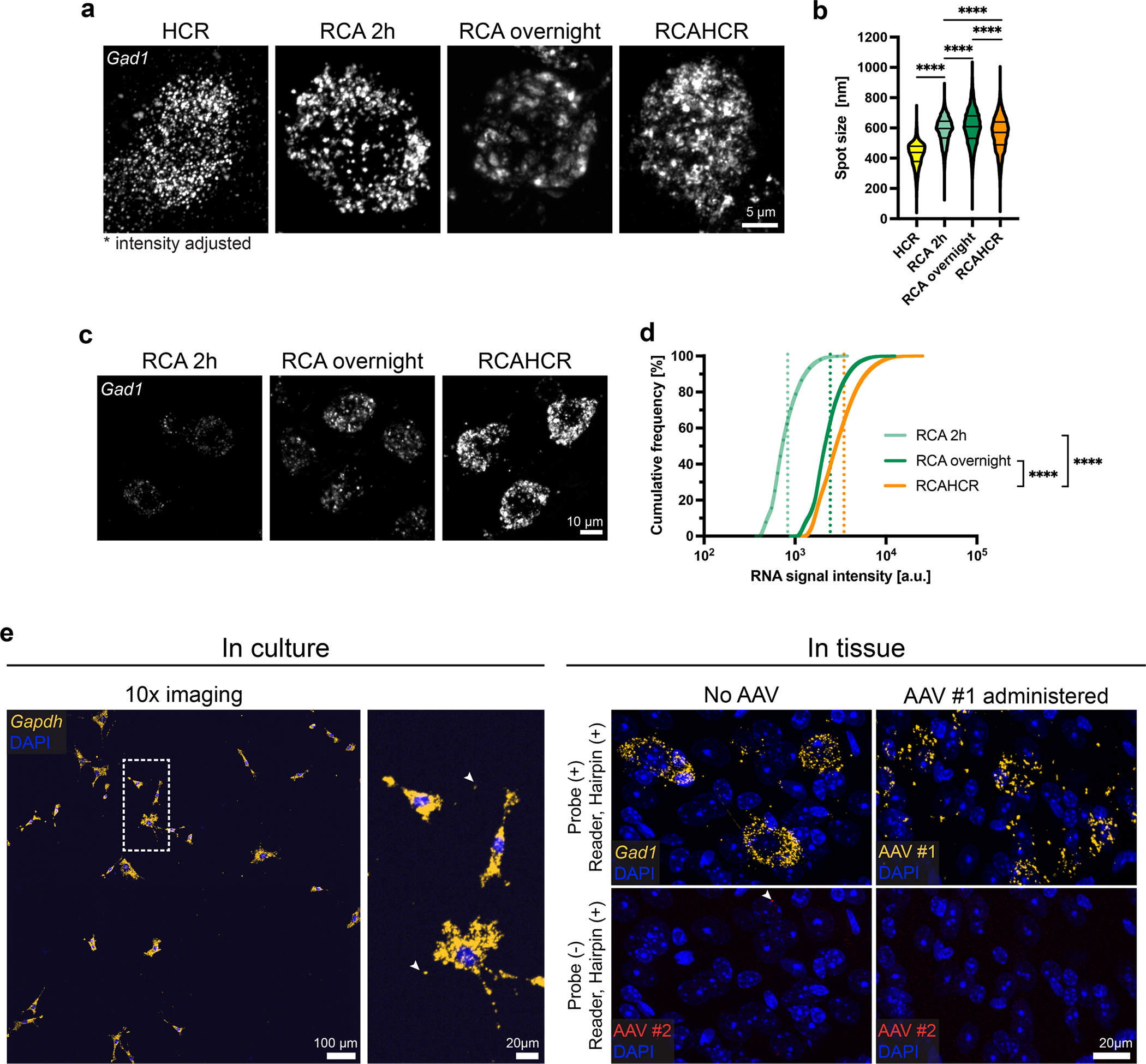
Further characterization of RCAHCR amplification. **a**, Representative images of *Gad1* in mouse brain tissue detected by HCR, RCA for 2 hours (RCA 2 h), RCA overnight, and RCAHCR. The intensity of each image was adjusted for better visualization. **b**, Quantification of spot sizes automatically detected by Imaris in each condition (n = 74,292 (HCR), 10,744 (RCA 2 h), 11,608 (RCA overnight), 24,798 spots (RCAHCR); ****p < 0.0001, two-sided MannWhitney test). **c**, Representative images of *Gad1* in mouse brain tissue detected by RCA 2 h, RCA overnight, and RCAHCR. The intensity range of all images was matched to show the different brightness of the signal. **d**, Cumulative distribution of RNA spot intensity of RCA 2 h, RCA overnight, and RCAHCR. Dashed lines indicate the mean of each distribution (****p < 0.0001, Kolmogorov-Smirnov test). **e**, The false-positive detection of RCAHCR in cell culture (left) and in tissue (right).

To assess the signal amplification performance of RCAHCR, we compared the intensity of RNA signals amplified with RCAHCR to those amplified with RCA-only or HCR-only in mouse brain tissue. To do so, we designed four probes against *Gad1* for each condition. For RCA-only amplification, we used the same probes designed for RCAHCR but added fluorophore-conjugated UGI* after RCA (as in STARmap^40^). For HCR-only amplification, we used four probes designed as split–initiator pairs as suggested for HCR version 3 (ref. ^54^) (HCR v3, Fig. 1c). In all cases, we used the same fluorophore (Alexa Fluor 647) to minimize color variance. We found that RCAHCR yielded an 11.7-fold and a 6.2-fold increase in mean signal intensity compared to HCR-only or RCA-only, respectively (Fig. 1d). RCAHCR also showed a significantly higher signal-to-background ratio (SBR; 10.9 ± 0.01) compared to HCR-only (1.39 ± 0.67) or RCA-only (3.09 ± 2.21; mean ± s.e.m.; Fig. 1e). Additionally, we confirmed that, compared to increasing the reaction time of RCA to overnight, RCAHCR (2 hours of RCA and subsequent 1 hour for HCR) produces RNA spots with similar size yet significantly higher intensity (Extended Data Fig. 1a–d). We also measured that the false positive of RCAHCR is 1 per 8,000 μm^2^ and 1 per 20,000 μm^3^ in cell culture and tissue, respectively (Extended Data Fig. 1e), which is much lower than HCR (1 per 3,000 μm^3^)^44^. Note that single-molecule FISH^56^ or other methods employing signal amplification (for example, RCA^48^, HCR^54^ or SABER^57^) usually recommend using at least 10–20 probes per target gene, and we were able to obtain a brighter RNA signal by adding more probes to HCR (‘13 probes’ in Fig. 1c). In contrast, RCAHCR uses only four probes, and, even with this small number, it generates much brighter and more sensitive RNA signals, which will be beneficial for targeting short endogenous sequences or designing short barcodes for viral genomes with a limited packaging capacity.

**Extended Data Fig. 2 |:**
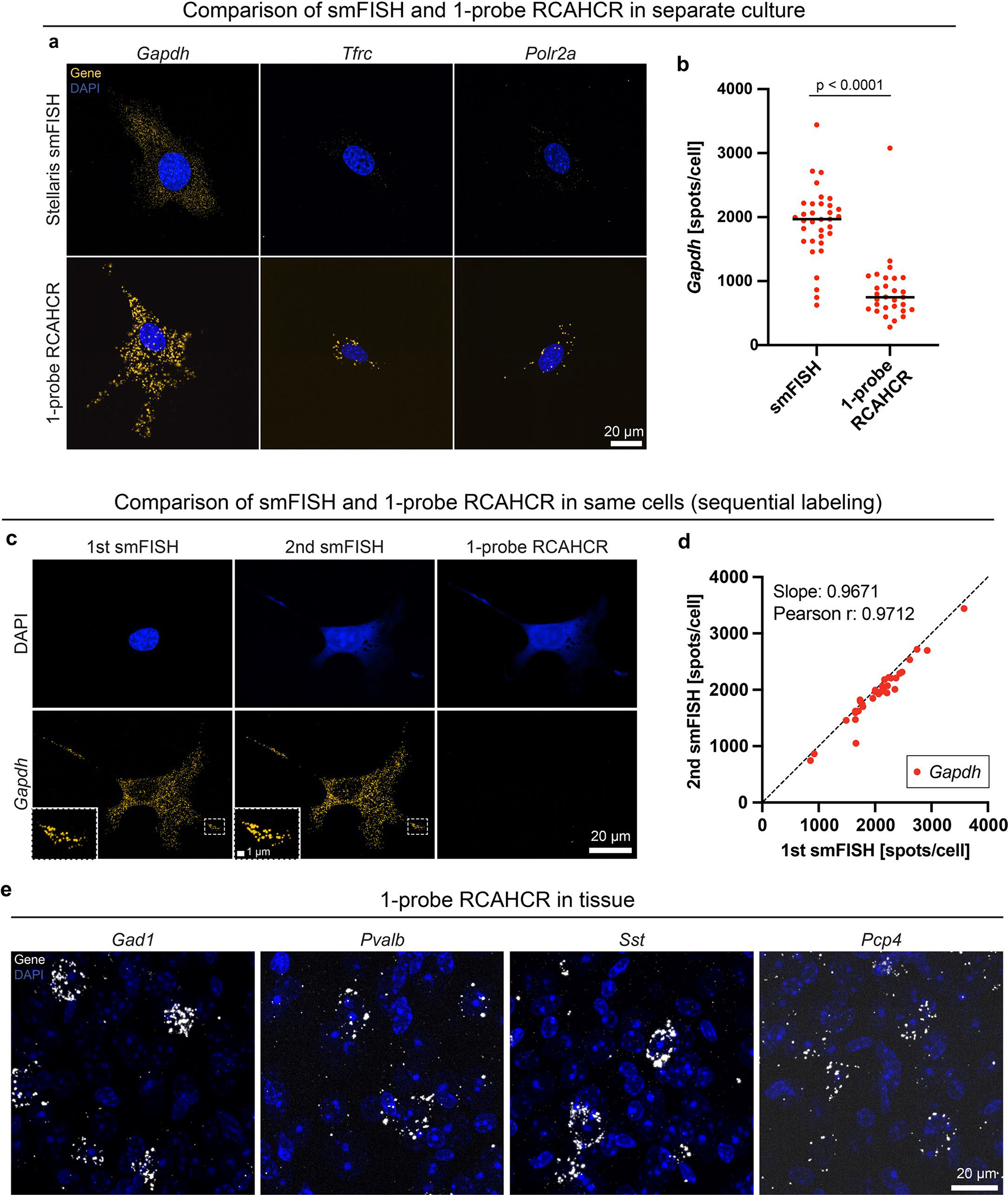
Further characterization of 1-probe RCAHCR. **a**, Representative images of three house keeping genes (*Gapdh*, *Tfrc*, and *Polr2a*) detected by smFISH and 1-probe RCAHCR in different NIH3T3 cells. **b**, Quantification of *Gapdh* spots/cells detected by smFISH and 1-probe RCAHCR (two-sided unpaired t-test). **c**, Representative images of *Gapdh* spots sequentially detected by 1^st^ smFISH, 2^nd^ smFISH, and 1-probe RCAHCR in the same NIH3T3 cells. The probe stripping was performed by DNase I treatment (0.5 U/μl). Note that the probe used for 1-probe RCAHCR is the same as in **a**. **d**, Quantification of 1^st^ and 2^nd^ smFISH spots in the same cells. The slope estimated from linear regression was 0.9671, and the Pearson correlation coefficient (r) was 0.9712. **e**, Representative images of *Gad1*, *Pvalb*, *Sst*, and *Pcp4* genes detected by 1-probe RCAHCR in mouse brain tissue.

Indeed, this remarkably high sensitivity of RCAHCR enabled RNA detection even with a single probe (a pair of a primer and a padlock; Fig. 1f–h). To validate the capability of single-probe detection with RCAHCR amplification, we assessed the detection efficiency of 1-probe RCAHCR compared to single-molecule HCR (smHCR) by measuring four housekeeping genes (*Gapdh*, *Eef2*, *Tfrc* and *Polr2a*) with various expression levels in the same NIH3T3 cells (Fig. 1f and Extended Data Fig. 2a–d)^42^. We found that the detection efficiency of 1-probe RCAHCR is 42–87% of smHCR with high correlation (Fig. 1g,h). We also confirmed that 1-probe RCAHCR can visualize endogenous genes in intact tissue volume (Extended Data Fig. 2e). Altogether, these results indicate that the method requires a unique sequence as short as 40 nt for selective endogenous RNA detection in tissue.

One caveat of RCAHCR is the limited penetration of the enzymes required for RCA that hinders three-dimensional (3D) tissue labeling (by comparison, HCR is capable of labeling tissue with a thickness of ~500 μm^58^). To address this, we reasoned that hydrogel-based tissue clearing could help the enzymes penetrate deeper into the tissue. By optimizing our passive CLARITY technique (PACT)^59,60^ to better retain mRNA transcripts without substantial interference in probe hybridization (Supplementary Fig. 1), we enabled multi-color detection of three endogenous genes in 50-μm-thick tissue using RCAHCR (Fig. 1i).

We next established a quenching method to increase the number of endogenous and viral genes that can be detected (Fig. 1a, ‘Two-step sequential labeling’). Most previous studies used DNase I to degrade hairpin assemblies^44,61^; however, this enzyme often leaves residual activity that can affect the next round of HCR and can also compromise the DNA amplicons. Thus, we instead used a hairpin with a toehold sequence (10 nt) that can induce spontaneous disassembly through strand displacement^41^. With these toehold hairpins, the two-step method is performed as follows. For the first round, we add a pair of toehold and fluorophore-conjugated hairpins to create the hairpin assembly. To dismantle the hairpin assembly, we add a short displacement oligo (a sequence complementary to the toehold and half of the hairpin). Next, formamide (60–70%) is used to detach the initiators from the amplicon to prevent undesired hairpin assembly during the next round of HCR. Once the signal is quenched, the next round of HCR can be initiated by adding another set of initiators, followed by HCR labeling.

**Extended Data Fig. 3 |:**
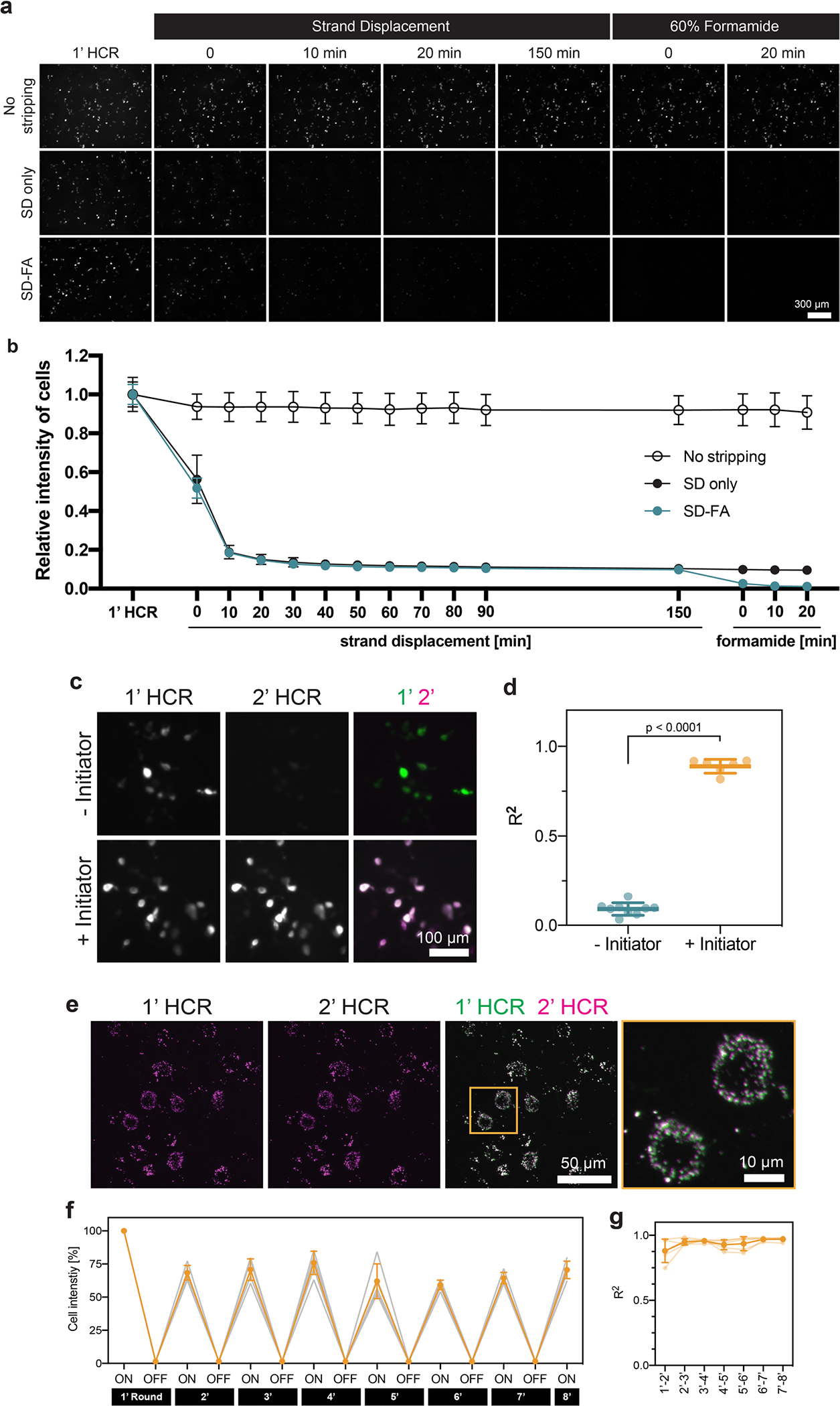
Validation of two-step stripping method. **a**, Representative images of the RNA signals of the barcode transfected into HEK293T cells and detected by RCAHCR (1’ HCR). Following 1’ HCR, we divided the samples into three groups: (1) no stripping, (2) only hairpin disassembly via strand displacement (SD only) and (3) hairpin disassembly via strand displacement and initiator detachment via formamide treatment (SD-FA). While proceeding with each step, we time-lapse imaged the samples after hairpin disassembly via strand displacement (‘Strand Displacement’), followed by initiator detachment with formamide (‘60% formamide’). **b**, Relative cell intensity of no-stripping, SD-only, and SD-FA samples in **a** after each step (n = 3; mean ± s.d.). **c**, Representative images of the RNA signals from the barcode after the first (1’ HCR) and second round of HCR (2’ HCR) with the same initiators and hairpins (+Initiator) or only the hairpins (−Initiator). **d**, The coefficient of determination (R^2^) between 1’ HCR and 2’ HCR in **c** (mean ± s.d.; two-sided unpaired t-test; n = 9 for ‘−Initiator’; n = 6 for ‘+Initiator’). **e**, In situ labeling of *Gad1* with RCAHCR amplification in mouse cortex (1’ HCR) and re-detection (2’ HCR) after two-step stripping. **f**, Relative cell intensity over eight rounds of the same barcode detection (used in **a**) in cell culture (n = 5; mean ± s.d.). **g**, The coefficient of determination (R^2^) between each round during the eight rounds of repeated labeling in **f** (n = 5; mean ± s.d.).

To examine whether our two-step stripping method could efficiently quench HCR signal, we transfected HEK293T cells with plasmids carrying a barcode (a unique 160-nt sequence orthogonal to the human transcriptome) and hybridized four probes complementary to this barcode. After RCA and the first round of HCR with the toehold hairpins, we compared cell intensity change over time in multiple conditions: ‘No stripping’ (no further steps), ‘SD only’ (displacement oligos only) and ‘SD-FA’ (both displacement oligos and subsequent formamide treatment) (Extended Data Fig. 3a,b). Treatment with both displacement oligos and formamide quickly and efficiently reduced cell intensity, suggesting near complete disassembly of hairpin structures.

We next asked whether our two-step method could prevent crosstalk between HCR rounds and preserve the DNA amplicon intact. If residual hairpins or initiators remained hybridized to amplicons, subsequent addition of hairpins could result in hairpin assembly at unwanted sequences, introducing substantial noise. Also, if the amplicons were damaged by the stripping method, further rounds of HCR would not be successful. To answer these questions, we attempted a second round of HCR on samples stripped by our method, using the same initiators and hairpins as for the first round (Extended Data Fig. 3c). When adding only hairpins without initiators, we were unable to trigger HCR assembly, indicating that residual hairpins and initiators are negligible after two-step stripping (‘−Initiator’ in Extended Data Fig. 3c,d). Using the same initiators and hairpins, we were able to recover HCR signals with R^2^ = 0.889 ± 0.016 (mean ± s.e.m.; ‘+Initiator’ in Extended Data Fig. 3c,d). Two rounds with the same hairpins and initiators for the same gene (*Gad1*) in tissue also highly overlapped (Extended Data Fig. 3e). Finally, we successfully achieved eight rounds of sequential labeling with the same hairpins and initiators in the same cell culture, preserving a mean R^2^ of >0.9 between rounds (Extended Data Fig. 3f,g). These results indicate that our two-step method facilitates sequential labeling with RCAHCR amplification, enabling the USeqFISH procedure.

We assessed the performance of USeqFISH in tissue. First, we observed no significant difference between the number of *Gad1* spots per cell detected with USeqFISH (four probes) and HCR (ref. ^54^), indicating that USeqFISH has a similar detection efficiency to HCR (~70%^44,54^; Fig. 1j and Supplementary Fig. 1b). We also compared the expression level of 26 endogenous genes in mouse cortex (Supplementary Table 1), measured as the mean spot count per cell with USeqFISH and as the mean unique molecular identifier (UMI) count per cell with scRNA-seq^32^. We found a significant linear correlation (*r* = 0.627; *P* = 6 × 10^−4^; Fig. 1k), with a higher detection efficiency of USeqFISH, especially for lower-abundance genes. The robustness of USeqFISH over multiple rounds was examined by detecting the same gene (*Gad2*) in the same cells at round 2 and round 13; ~76% of the signal was preserved (Fig. 1l). Taken together, our results show that USeqFISH can detect ~50 RNAs (4 colors × 13 rounds) in intact tissue volumes by targeting ≤160 nt of each sequence without substantial loss (compared to HCR or scRNA-seq) yet with much brighter signal.

**Fig. 2 |:**
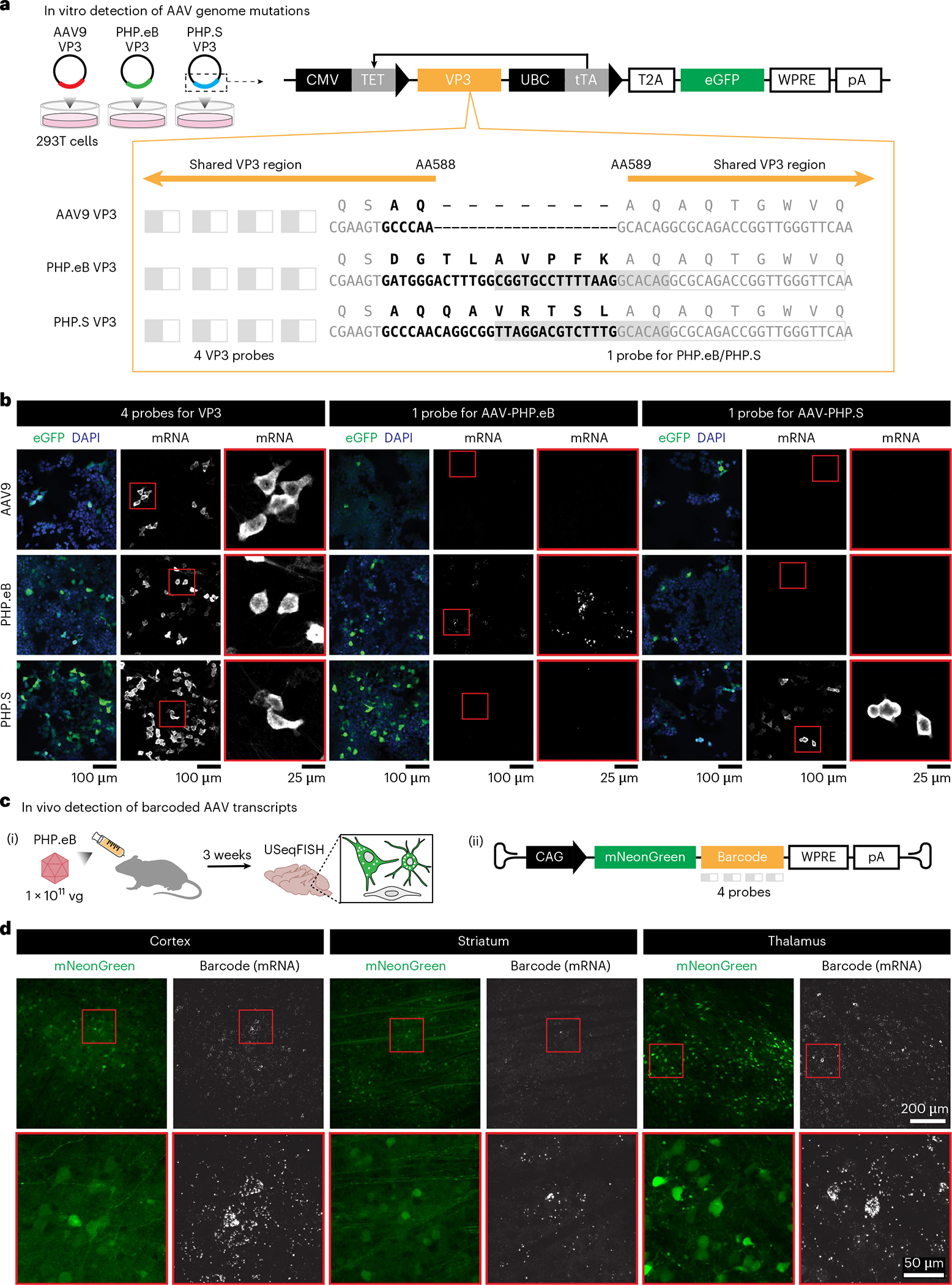
High sensitivity of USeqFISH detects short mutations and barcodes in the AAV genome in vitro and in vivo. **a**, Three plasmids were designed to carry the VP3 of AAV9, AAV-PHP.eB (‘PHP.eB’) and AAV-PHP.S (‘PHP.S’) with eGFP. AAV-PHP.eB and AAV-PHP.S have distinct 9-AA and 7-AA mutations (bold letters) in the same location (AA588) of the AAV9 VP3 sequence. After transfecting into HEK293T cells, we detected the transcripts of each plasmid using the following probes (gray filled boxes indicate the padlock target sequence, and gray outlined boxes indicate the primer target sequence): four probes against the shared VP3 sequence, one probe against the insertion of AAV-PHP.eB and one probe against the insertion of AAV-PHP.S. For the probes against each insertion, we used the same primers for AAV-PHP.eB and AAV-PHP.S but distinct padlocks that differed by 14 nt. **b**, Detection of the VP3 transcripts with four probes for VP3, one probe for AAV-PHP.eB and one probe for AAV-PHP.S in HEK293T cells expressing the VP3 of AAV9, AAV-PHP.eB and AAV-PHP.S. **c**, For in vivo detection, we designed a viral genome carrying mNeonGreen and a barcode and systemically delivered it to adult mice using AAV-PHP.eB at a dose of 1 × 10^11^ vg per mouse. At 3 weeks after injection, we used USeqFISH with probes against the barcode to detect viral transcripts in tissue. **d**, Detection of viral barcodes (‘Barcode (mRNA)’) in cells expressing mNeonGreen (green) in various mouse brain regions (cortex, striatum and thalamus).

We next investigated whether USeqFISH could detect RNAs transcribed from viral genomes transfected into cultured cells (Fig. 2). To do so, we designed a plasmid encoding the AAV9 VP3 protein with an eGFP tag. We then cloned amino acid (AA) mutations into this plasmid to generate the VP3 of AAV-PHP.eB^6^ (a 2-AA substitution and a 7-AA insertion) and AAV-PHP.S^6^ (a 7-AA insertion) at AA588 of AAV9. After transfection of each plasmid into HEK293T cells, we applied USeqFISH with three different probe sets: four probes against the VP3 sequence shared across all three variants, one probe against part of the 7-AA insertion of AAV-PHP.eB and one probe against part of the 7-AA insertion of AAV-PHP.S (Fig. 2a). Note that, for the probes targeting the insertions, only the padlocks differed by 14 nt, and the same primer was shared. With these VP3 probes, we were able to label the transcripts of all three plasmids in the cells expressing eGFP. With the single probe for each insertion, only the cells transfected with the corresponding plasmid were labeled (Fig. 2b), indicating that USeqFISH can selectively detect a mutated region in the viral genome as short as 14 nt in vitro.

We also tested whether USeqFISH could detect transcripts from the AAV genome in tissue after systemic delivery (Fig. 2c). We produced AAV-PHP.eB packaging pAAV-CAG-mNeonGreen (mNG)-WPRE-hGHpA with a barcode inserted between mNG and WPRE; this location facilitated barcode detection (Supplementary Fig. 2a). We intravenously (IV) administered the virus to adult mice at a dose of 1 × 10^11^ vector genomes (vg) per animal and harvested the brains at 3 weeks after injection. Using USeqFISH with probes against the barcode, we were able to label the RNA transcripts of the viral genome in cells expressing mNG from various brain regions (Fig. 2d). These results support the applicability of USeqFISH for parallel detection of multiple systemically delivered AAVs in tissue by reading barcodes uniquely assigned to each viral genome.

### In situ profiling of pooled systemic AAVs at various doses

**Extended Data Fig. 4 |:**
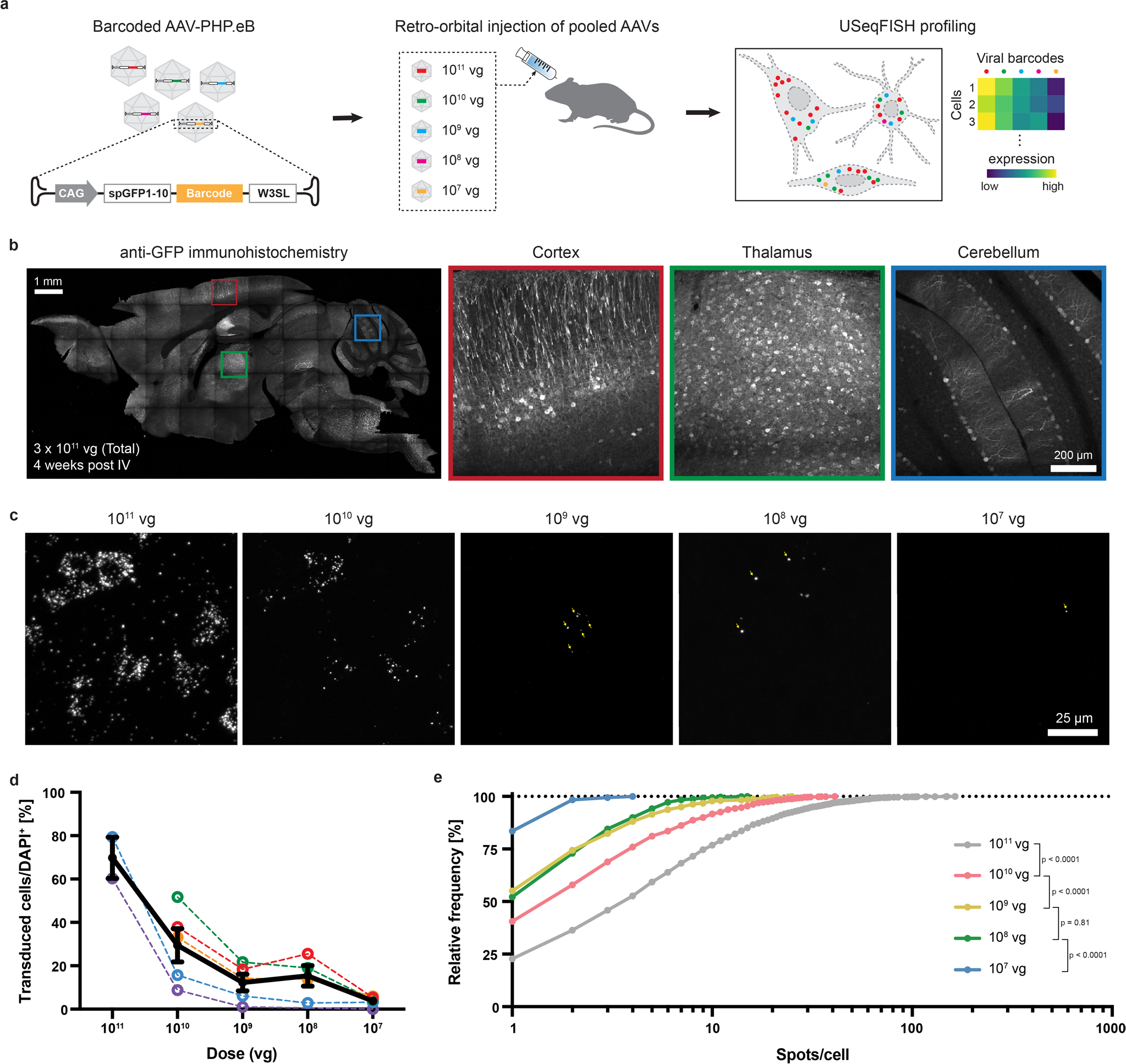
In situ dose-dependent transduction profiling of barcoded AAV pools with USeqFISH. **a**, Schematic of experimental design. A pool composed of AAV-PHP.eB carrying the viral genome with five unique barcodes was delivered at different doses (10^11^, 10^10^, 10^9^, 10^8^ and 10^7^ vg for each barcode per animal). After delivering the pool to mice via retro-orbital injection, USeqFISH was applied to the harvested brain tissue to detect the barcodes of each variant in parallel. **b**, Immunohistochemistry (IHC) with GFP antibodies, labeling spGFP(1–10) used as the coding sequence of the barcoded viral genome, showed broad expression of the injected pool across brain regions, including the cortex (red outlined box), thalamus (green outlined box), and cerebellum (blue outlined box). **c**, Representative images of the expression of the 5-dose pool. Viral transcript spots are indicated with yellow arrows in the images of lower doses (10^9^, 10^8^, and 10^7^ vg). **d**, The transduction rate of AAV-PHP.eB at each dose acquired from five mice (indicated by different colors; the mean of each dose is indicated by the black line; mean ± s.e.m.). **e**, The cumulative distribution of viral RNA spot number in each transduced cell at each dose (Kolmogorov-Smirnov test).

To apply USeqFISH for in situ profiling of pooled AAVs, we further designed the viral cargo to include (1) a non-fluorescent, short coding sequence and (2) a shortened WPRE, W3SL^62^, making the essential components as compact as possible to leave room for the sequences to be tested (Extended Data Fig. 4a). For the coding sequence, we used the first part of split GFP (spGFP(1–10)^63^) owing to its short length (642 base pairs (bp)) and its ability to be labeled by GFP antibodies for purposes of viral injection and expression quality control (Extended Data Fig. 4b). Then, we cloned a uniquely designed barcode (160 nt in length for four probes) into the backbone between spGFP(1–10) and W3SL. We confirmed that the barcodes inserted into the viral genome can be selectively detected only with the complementary probes in vitro (Supplementary Fig. 2b).

Because the total dose for systemically administered AAVs is limited (~10^12^ vg per adult mouse) mainly due to liver toxicity^27^, pooled administration requires each variant to be delivered at a lower dose. To determine the minimum dose that USeqFISH can detect, we designed a cocktail of the same AAV-PHP.eB carrying five unique barcodes delivered at different doses (10^11^, 10^10^, 10^9^, 10^8^ and 10^7^ vg per variant per animal) to adult wild-type (WT) mice (*n* = 5) via IV administration. Three weeks after injection, we harvested the brains and applied USeqFISH to detect all barcodes expressed in the mouse cortex (Extended Data Fig. 4c). Considering cells expressing at least one viral RNA spot to be transduced, we measured the transduction rate of AAV-PHP.eB at each dose. The transduction rate at the dose of 10^11^ vg was ~70%, similar to that observed by Chan et al.^6^, but dropped to ~4% at the dose of 10^7^ vg (mean; Extended Data Fig. 4d). In addition to the transduction rate, the distribution of viral transcript numbers per transduced cell showed that higher doses were associated with higher spot counts, suggesting that high-dose administration could lead to more cells being transduced as well as more AAV-delivered transgenes being expressed in each cell (Extended Data Fig. 4e). Collectively, these results suggest that the efficiency of both transduction and expression of the transgene packaged in systemic AAVs (here, AAV-PHP.eB) relies on the dose injected and underscore the importance of using a matched dose across experimental batches for accurate validation and characterization of AAV capsids. Our results also show that USeqFISH can detect AAV transcripts even at the minimum dose of 10^7^ vg; however, because <20% of cells were transduced and expressed only a few spots at doses of 10^9^ vg or lower, we conclude that, for quantitative analysis, a dose of ≥10^10^ vg for each variant would be required.

### In situ cell type tropism profiling of pooled systemic AAVs

**Fig. 3 |:**
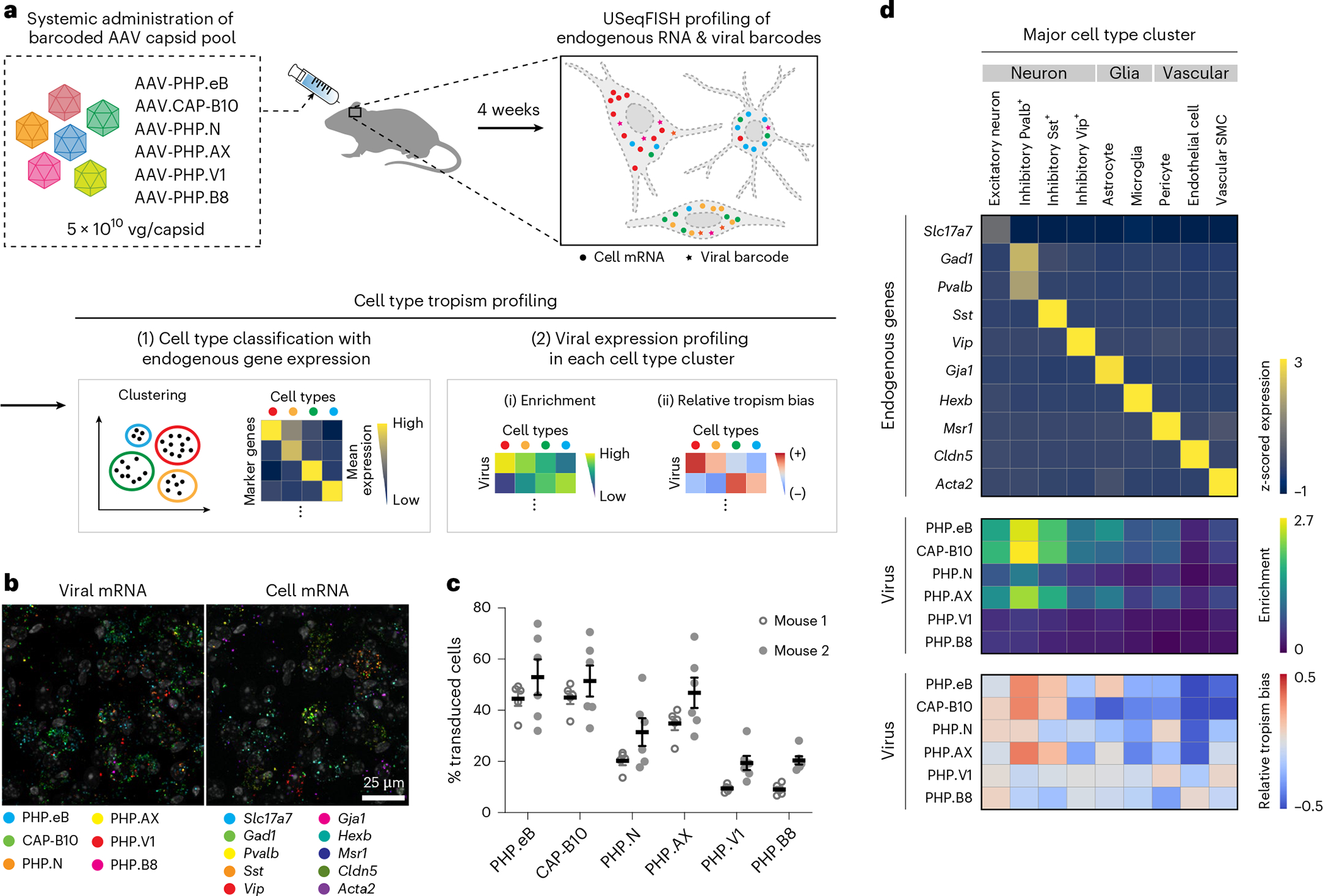
In situ major cell type tropism profiling of barcoded systemic AAV capsid pools in mouse cortex. **a**, Experimental pipeline. We designed a pool of six AAV capsid variants carrying unique barcodes and administered them to adult wild-type mice through retro-orbital injection. At 4 weeks after injection, we harvested the brain tissue and used USeqFISH to profile viral gene expression along with endogenous cell type marker genes. The image dataset was then converted to a gene-by-cell expression matrix via our automated image processing pipeline, and we quantitatively analyzed the data by clustering endogenous genes to identify cell type clusters, followed by viral gene expression profiling in each cluster. **b**, Representative image of six variants and ten cell type markers in the same region of the mouse cortex. **c**, Transduction efficiency, measured by % transduced cells, of each variant in two mice (mean ± s.e.m.; *n* = 5 for mouse 1; *n* = 6 for mouse 2). **d**, Endogenous (top, cividis color map) and viral gene expression profiles (enrichment: middle, viridis; relative tropism bias: bottom, coolwarm) in the cell type clusters.

To demonstrate the capability of USeqFISH for high-throughput, high-resolution profiling of AAVs, we designed a pool of six systemic AAVs (Fig. 3a). This pool includes previously identified capsids (AAV-PHP.eB^6^, AAV.CAP-B10 (ref. ^8^), AAV-PHP.N^7^, AAV-PHP.V1 (ref. ^7^) and AAV-PHP.B8 (ref. ^7^)) that show a range of efficiency and specificity of mouse CNS transduction across the blood–brain barrier (BBB). We also included a new capsid, AAV-PHP.AX, in the same pool to test if USeqFISH profiling can enable deep characterization of previously unexplored variants.

**Extended Data Fig. 5 |:**
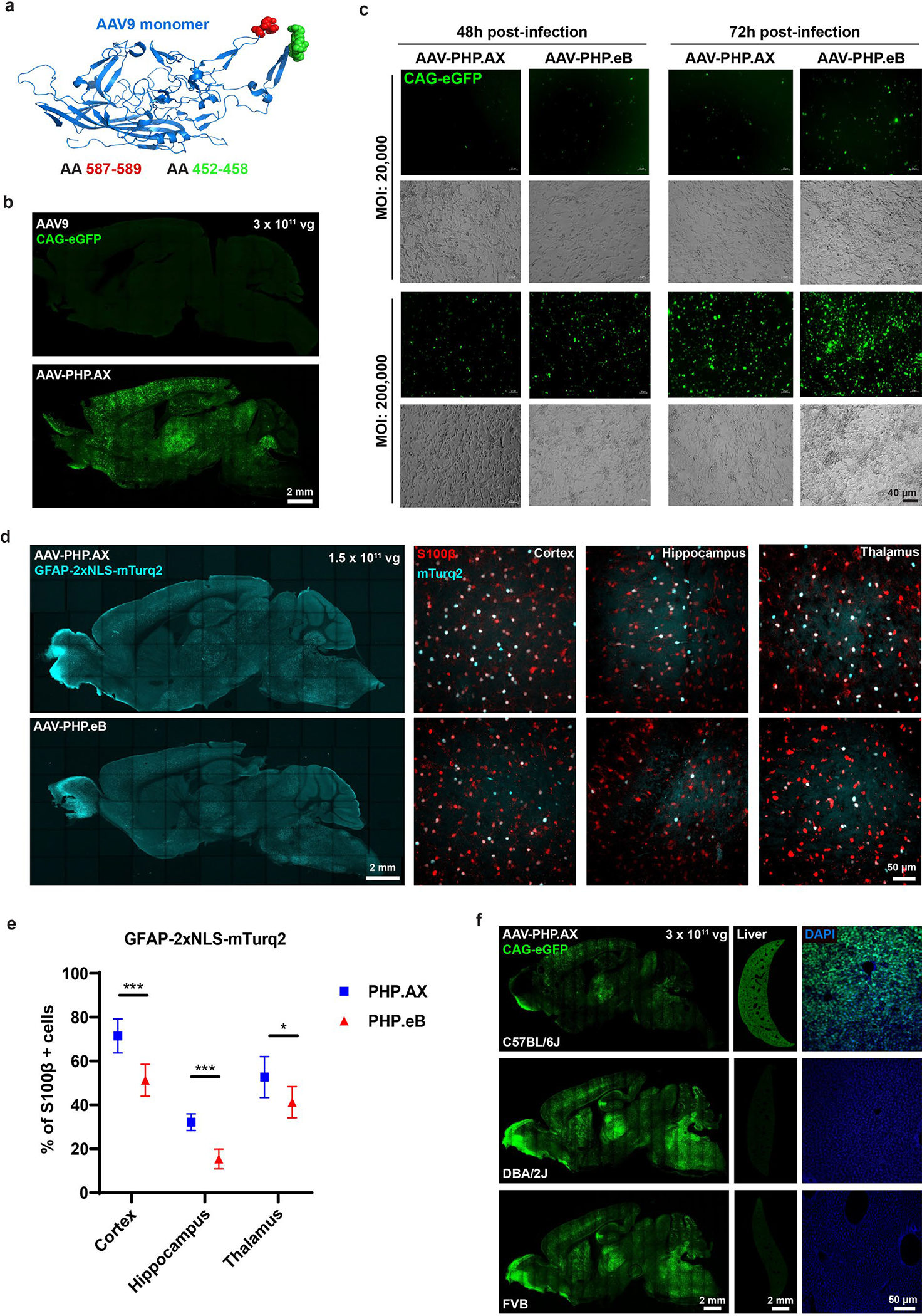
AAV-PHP.AX characterization following single injection in mouse brain. **a**, AAV-PHP.AX was engineered by substituting a 7-amino acid (AA) sequence of a glial homing peptide (green), at AA452–458 of AAV-PHP.eB (9-AA peptide shown in red). **b**, AAV9 and AAV-PHP.AX (each packaging a single-stranded CAG-eGFP genome) were delivered by retro-orbital injection to 8-week-old C57BL/6 J male mice (n ≥ 3 per group) at 3 × 10^11^ vg/mouse. Transgene expression was evaluated three weeks later. **c**, Representative images of human U87 glioblastoma (astrocyte-like) cells transduced by AAV-PHP.AX or AAV-PHP.eB, packaging CAG-eGFP, 48 or 72 hours post-infection (n ≥ 3 wells per condition). **d**, **e**, AAV-PHP.AX transduces astrocytes more efficiently than AAV-PHP.eB when paired with an astrocytic promoter. AAV:GFAP-2xNLS (nuclear localization signal)-mTurq2 was delivered by retro-orbital injection to 8-week-old C57BL/6 J male mice (n = 3 per group) at 1.5 × 10^11^ vg/mouse. Transgene expression was evaluated three weeks later. **d**, Brain sections were stained with the astrocyte marker S100β (red). Representative images of the cortex, hippocampus, and thalamus are shown. **e**, Quantification of the percentage of S100β + cells transduced by AAV-PHP.AX and AAV-PHP.eB (mean ± SD; two-way ANOVA, Tukey’s multiple comparisons test with adjusted p values; ***p = 0.0001 for cortex; ***p = 0.0006 for hippocampus; *p = 0.0217 for thalamus). **f**, AAV-PHP. AX efficiently transduces the brain after intravenous administration to C57BL/6 J, DBA2/J, and FVB mice. AAV-PHP.AX:CAG-2xNLS-eGFP was delivered by retro-orbital injection to 8-week-old male mice (n ≥ 5 per group) at 3 × 10^11^ vg/mouse. Transgene expression was evaluated three weeks later. Representative images of the brain and liver are shown. DAPI staining for nuclei is shown in blue.

AAV-PHP.AX is a variant rationally designed to display a tropism-homing peptide (NTGSPYE, shown to target microglia^55^) by substituting AA452–458 of AAV-PHP.eB (Extended Data Fig. 5). In a previous study, we successfully derived an efficient neurotropic capsid, AAV.CAP-B10, by screening a 7-AA substitution library of the highly protruding AA455 loop in AAV-PHP.eB^8^. This result demonstrated that it was possible to ‘add’ specificity by introducing diverse mutations to the capsid and motivated us to engineer the same location of AAV-PHP.eB with previously identified homing peptides.

**Extended Data Fig. 6 |:**
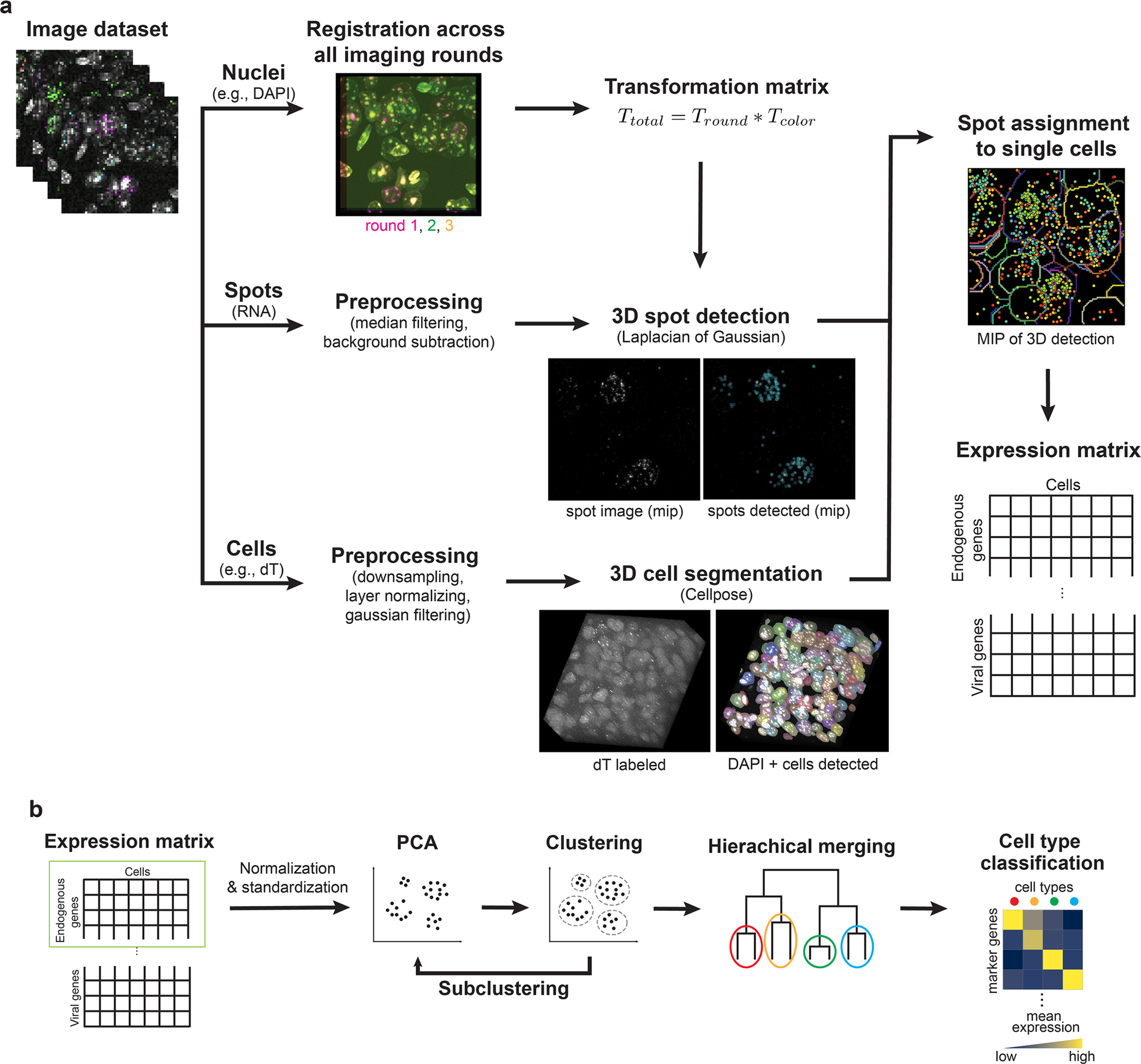
Automated 3D image processing and quantitative data analysis pipeline for USeqFISH. **a**, A collection of volume imaging data for each USeqFISH experiment consists of nuclei labeling and RNA spots for each round and cytosolic labeling for the last round. Using the nuclei labeling for each round, we calculated the rigid transformation matrix to the last image for registration across imaging rounds. We combined this transformation matrix with one for correcting optical aberration obtained with fluorescent microbeads prior to the experiment. For RNA spot detection, we proceeded with smoothing by 3-pixel median filtering and background subtraction for each volume and applied a Laplacian of Gaussian filter to obtain the location of each spot. For cell body segmentation, we pre-processed the dT labeled image and used it to identify single cells by applying Cellpose. These three calculations were then combined to register all volumes into the same coordinates, to assign each spot to each cell, and finally to obtain the cells-by-genes expression matrix. **b**, The expression matrix of endogenous genes was then normalized and z-standardized and used to cluster cell types with endogenous genes by applying principal component analysis (PCA), followed by Leiden clustering. The viral gene expression profiles were analyzed along the clusters identified (details in Methods and Fig. 3a).

Using the same backbone as in the dose-dependency study, we individually produced the viruses to guarantee that each one packaged a unique barcode, and we IV administered the pool to adult WT mice (*n* = 2) at a dose of 5 × 10^10^ vg per variant (for a total dose of 3 × 10^11^ vg per animal). We harvested the brains after 4 weeks of expression and applied USeqFISH with probe sets for the barcodes of each variant and for canonical cell type marker genes that we selected from previously published scRNA-seq studies^43,64–68^. By applying our computational image processing and data analysis pipeline, we obtained an expression matrix of endogenous genes and viral barcodes (Extended Data Fig. 6a). With cell type clusters identified from the endogenous gene expression matrix (Methods), we analyzed the expression of the viral barcodes in each cell type cluster (Extended Data Fig. 6b). First, to assess the transduction efficiency of all variants in the pool across cell types, we measured the enrichment (mean of log-transformed spot numbers) of each variant in each cell cluster. Second, to compare relative cell type tropism bias across variants, we measured z-scored spot numbers of each barcode log-normalized to the total barcode counts per cell, with the hypothesis that the ratio between variants’ transcript numbers would be conserved across all cell types if all variants have the same tropism.

We first asked whether multiplexed analysis of these six AAVs with USeqFISH could recapitulate our previously reported characterization results from multiple studies with IHC^6–8^ or scRNA-seq^32^. To do so, we analyzed a total of 4,330 cells in the cortex of two mice using ten marker genes that represent cell types known to be preferred or excluded by each capsid, including neurons (*Slc17a7* for pan-excitatory neurons; *Gad1* for pan-inhibitory neurons; and *Pvalb*, *Sst* and *Vip* for major inhibitory neuronal subtypes), glia (*Gja1* for astrocytes and *Hexb* for microglia) and vascular cells *(Msr1* for pericytes, *Cldn5* for endothelial cells and *Acta2* for vascular smooth muscle cells (SMCs)) (Fig. 3b and Supplementary Fig. 3). Although a direct comparison to previous results would be inappropriate owing to the different doses used, the trend of overall transduction efficiency across the variants conformed to our expectations (Fig. 3c): AAV-PHP.eB and AAV.CAP-B10 showed similar transduction efficiency (~48%) and much higher than other variants^8^. The overall transduction efficiency of AAV-PHP.N was modest (~26%) and that of AAV-PHP.V1 and AAV-PHP.B8 was low (~14%) due to their expected tropism bias toward vascular cells and thalamus/cerebellum, respectively^7^. The new variant, AAV-PHP.AX, showed slightly lower transduction efficiency (~41%) than the most efficient ones (AAV-PHP.eB and AAV.CAP-B10) but much higher than the others.

Through USeqFISH profiling, we identified nine cell type clusters in which each cell type marker gene is highly expressed, and we assessed the distribution of each variant across these cell types (Fig. 3d). We found that the most efficient variants (AAV-PHP.eB, AAV.CAP-B10, AAV-PHP.N and AAV-PHP.AX) showed strong enrichment in neurons, particularly in *Pvalb*^+^ inhibitory cells (presumably due to the slight inhibitory bias of the CAG promoter^69^), and lower enrichment in *Vip*^+^ inhibitory cells, consistent with scRNA-seq results^32^. All six variants exhibited lower enrichment in non-neuronal cells than in neurons. In previous IHC studies, some variants showed higher transduction rates in non-neuronal cells; for example, AAV-PHP.V1 transduced astrocytes (S100+: ~60%) and endothelial cells (Glut1+: ~60%) more efficiently than neurons (NeuN+: 10%)^7^. This discrepancy could be due to the different markers and doses used in this study or to underestimation of non-neuronal cells by USeqFISH (Discussion). Nonetheless, USeqFISH showed higher enrichment of AAV-PHP.eB than AAV.CAP-B10 in astrocytes, supporting the relative neuronal preference of AAV. CAP-B10 (ref. ^8^).

Overall, our relative tropism analysis was consistent with our previous results. Again, AAV-PHP.eB showed relative tropism bias toward inhibitory neurons and astrocytes, whereas AAV.CAP-B10 showed bias toward neurons (both excitatory and inhibitory) and away from astrocytes^8,32^. AAV-PHP.N was relatively neuronal^7^, with lower transduction efficiency than AAV.CAP-B10. Although much less enriched than the other capsids, AAV-PHP.V1 showed relative tropism toward vascular cells (pericytes and vascular SMCs) as expected^7^. Collectively, these results show that USeqFISH can characterize the transduction and tropism of pooled AAVs with high throughput. In addition, the endogenous gene-based cell type clustering and transduction profiles of the six variants were strongly conserved between the two mice (Supplementary Fig. 4), supporting the reproducibility of USeqFISH-based AAV profiling.

**Fig. 4 |:**
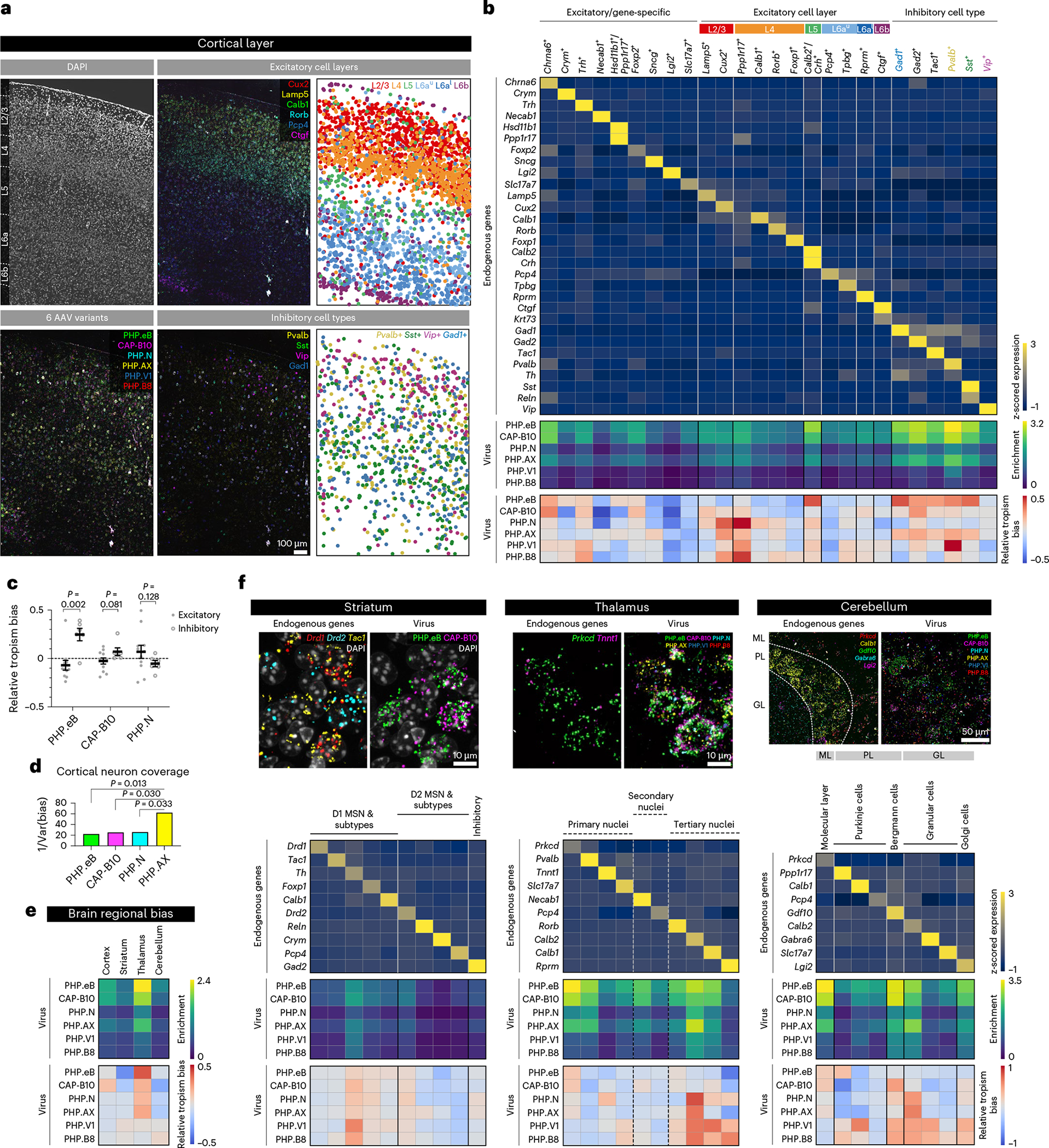
Neuronal subtype tropism profiling of systemic AAVs in mouse cortical layers and other brain regions. **a**, Labeling of the cortex region by DAPI and the six AAV variants. For excitatory cell layers and inhibitory subtypes, the left and middle panels show the real RNA images of selected genes acquired from the experiment, and the right panels show the cell types inferred from clustering based on endogenous gene expression. **b**, Endogenous (top, cividis color map) and viral gene expression profiles (enrichment: middle, viridis; relative tropism bias: bottom, coolwarm) across the cell type clusters identified. Colored cell types are visualized in **a**. **c**, The relative tropism bias of AAV-PHP.eB, AAV.CAP-B10 and AAV-PHP.N across the group of excitatory neurons (total 11 clusters) and inhibitory neurons (total six clusters; mean ± s.e.m.; two-sided unpaired *t*-test). **d**, The cortical neuron coverage of efficient variants (AAV-PHP.eB, AAV.CAP-B10, AAV-PHP.N and AAV-PHP.AX) measured by the inverse variance of relative tropism bias (the coolwarm heat map in **b**) across all cell type clusters (*F*-test on variance). We omitted AAV-PHP.V1 and AAV-PHP.B8 from this analysis as their transduction efficiency is too low to be considered for overall neuronal transduction. **e**, Viral expression profiles across selected mouse brain regions (cortex, striatum, thalamus and cerebellum). We separated the endogenous gene expression matrix into the fields of view of each region and used this profile to identify the regional bias of the variants. **f**, Endogenous and viral gene expression profiles in cell type clusters identified in the striatum, thalamus and cerebellum. We selected ten genes for striatum, ten for thalamus and nine for cerebellum that have been shown to be enriched in each region and classified cells into the clusters represented by each gene. Based on their Ward distance dendrogram, we manually merged clusters into known subtypes. Unlike the striatum and cerebellum, which are composed of genetically distinct cell types, the thalamus has relatively gradual variation in gene expression across topographical nuclei; therefore, we separated cells into three putative groups (marked by dashed lines).

For more in-depth characterization, we next investigated the enrichment and relative tropism of our variant pool across neuronal subtypes in mouse cortical layers using 30 cell type/layer-specific markers (a total of 8,475 cells were analyzed in a 1.14 mm × 1.69 mm area; Fig. 4a–d). Based on our selected gene markers, we classified cells into 26 clusters, including nine excitatory/gene-specific, 11 excitatory/layer-specific and six inhibitory subtypes (Fig. 4a,b and Supplementary Fig. 5). Using these cell type clusters, we identified a few interesting features of the variants (Fig. 4b). The overall enrichment pattern—AAV-PHP.eB and AAV.CAP-B10 highly enriched, followed by AAV-PHP. AX and AAV-PHP.N and then low-efficiency AAV-PHP.V1 and AAV-PHP. B8—was consistent with our previous results, as was the variants’ bias toward inhibitory neurons with a lower preference for *Vip*^+^ cells. Despite similar enrichment patterns, AAV-PHP.eB and AAV.CAP-B10 differed in their tropism, with AAV-PHP.eB being relatively biased toward L5 and inhibitory neurons and AAV.CAP.B10 showing relative bias toward L2/3 and L4. Interestingly, despite a lower transduction efficiency than the most efficient variants (AAV-PHP.eB and AAV.CAP-B10), AAV-PHP.N was relatively biased toward excitatory neurons. When the biases of these three variants were compared between excitatory and inhibitory neuronal clusters, AAV-PHP.eB was significantly biased toward inhibitory neuronal subtypes. Although AAV.CAP-B10 showed slight bias toward inhibitory subtypes, AAV-PHP.N was biased toward excitatory subtypes (Fig. 4c).

Another interesting result is that, compared to other variants, AAV-PHP.AX showed relatively unbiased and broad coverage (measured by the inverse of bias variance) across neuronal subtype clusters identified (Fig. 4d). Given its relatively robust transduction efficiency and enrichment in astrocytes (Fig. 3d), this variant could potentially serve as a high-efficiency universal vector that can be paired with cell-type-specific gene regulatory elements for targeted transduction. In fact, despite showing less enrichment in astrocytes than AAV-PHP.eB with a ubiquitous promoter (Fig. 3d and Extended Data Fig. 5c), with the astrocyte-specific glial fibrillary acidic protein (GFAP) promoter, AAV-PHP.AX more efficiently transduced astrocytes than AAV-PHP.eB delivering the same cargo (Extended Data Fig. 5d,e). These results suggest that AAV-PHP.AX has a higher capacity for tropism modulation by engineered cargos. Collectively, our results show that USeqFISH can provide transduction and tropism profiles of AAVs at the cell subtype level, in addition to facilitating inter-variant comparison in the same animal.

Broadening our coverage to other brain areas, we further sought to determine the cell type tropisms of our AAV pool in mouse striatum, thalamus and cerebellum (Fig. 4e,f). From sagittal sections, we collected 4–6 fields of view of each region (including the cortex as a control) and pooled all the data for quantitative analysis (6,929 cells were analyzed in total). We first examined the enrichment and relative tropism of each variant across the regions regardless of cell type to see whether our approach could recapitulate prior observations of overall and region-biased expression patterns (Fig. 4e). Our results show that the thalamus was the most favorable among the four regions for all six capsids, as expected. Note that, compared to other variants, AAV. CAP-B10 was highly biased to cortex yet largely away from cerebellum, consistent with our previous observation^8^. Although slightly more enriched in thalamus than other regions, we discerned no noticeable regional bias for AAV-PHP.V1 and AAV-PHP.B8 compared to other variants, presumably due to the lower dose used in our pool and the lower transduction efficiency of both variants.

**Extended Data Fig. 7 |:**
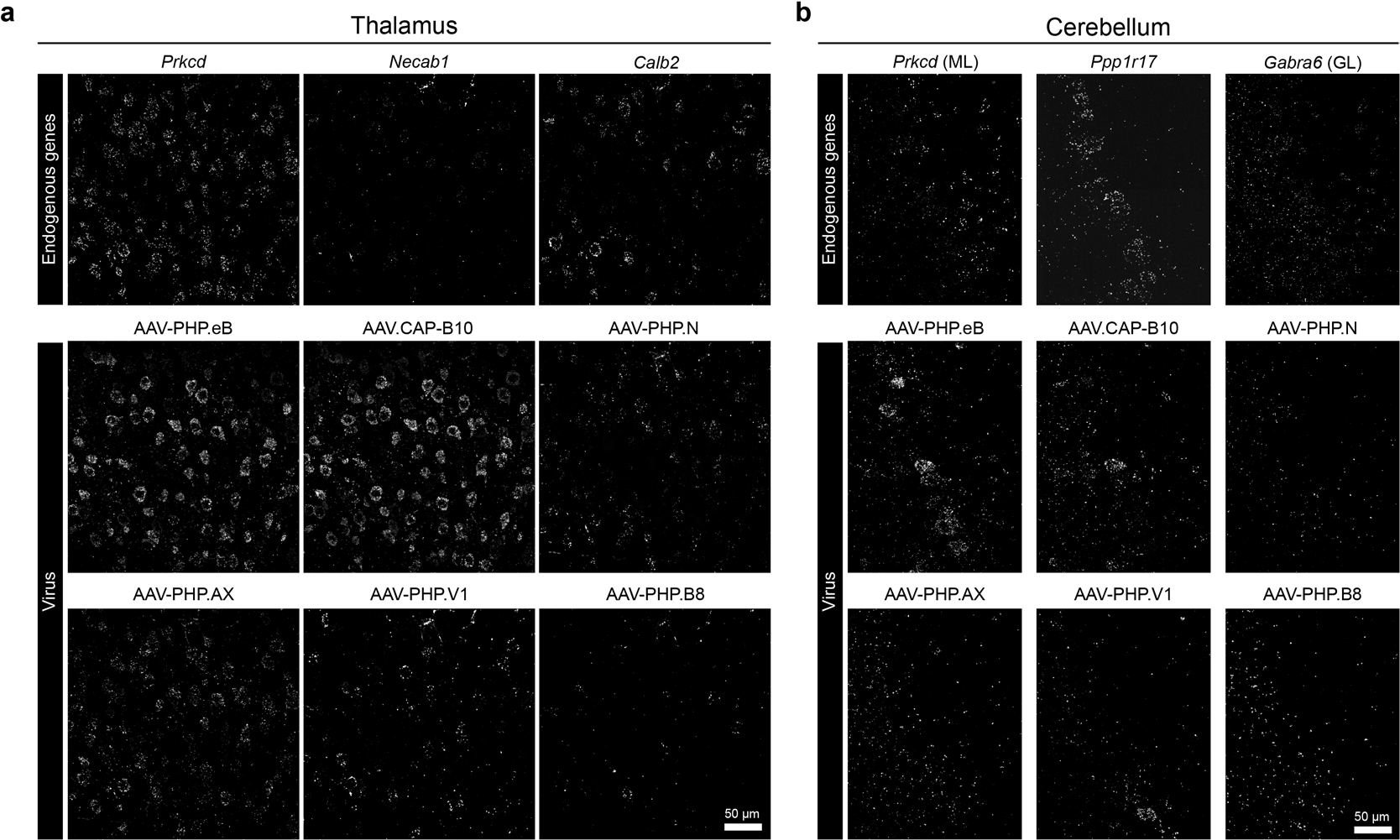
Examples of endogenous and AAV transcripts in mouse thalamus and cerebellum. Representative RNA images of endogenous cell-type marker genes (*Prkcd*, *Necab1*, and *Calb2* for thalamus; *Prkcd*, *Ppp1r17*, and *Gabra6* for cerebellum) and six variants in the same region of the mouse thalamus (**a**) and cerebellum (**b**).

Next, we profiled our AAV pool across major neuronal subtypes in each brain region. We selected ten cell type marker genes enriched in the striatum^66^, ten in the thalamus^68^ and nine in the cerebellum^67^ (Fig. 4f). We then identified cell type clusters represented by these individual marker genes and manually merged them into known groups based on Ward distances between clusters. Note that, whereas the striatum and the cerebellum are comprised of genetically distinct cell types, thalamic cell profiles have been shown to be rather continuous along topographically organized nuclei^68^; therefore, we split the clusters into three major groups based on markers (*Tnnt1* for primary, *Necab1* for secondary and *Calb2* for tertiary nuclei)^68^, and this putative separation is marked by dashed lines in Fig. 4f. In the striatum (total 2,010 cells), we found that all six variants showed similar enrichment and relative tropism. Most variants transduced both D1 and D2 medium spiny neurons (MSNs) as well as *Gad2*^+^ inhibitory cells (with a slight preference for *Th*^+^ cells). In the thalamus (total 1,481 cells) and cerebellum (total 1,428 cells), on the other hand, they transduced most region-specific cell types with slightly distinct preferences. In the thalamus, AAV-PHP.eB and AAV. CAP-B10 were highly biased toward *Prkcd*^+^ cells, whereas AAV-PHP.N and AAV-PHP.AX preferred *Calb2*^+^ cells (Fig. 4f and Extended Data Fig. 7a). In the cerebellum, AAV-PHP.eB showed a bias toward Purkinje cells in the Purkinje layer (PL) and the molecular layer (ML), whereas AAV-PHP.N and AAV-PHP.AX were biased toward the granular layer (GL) (Fig. 4f and Extended Data Fig. 7b). Despite low overall enrichment, AAV.CAP-B10 showed relative bias toward ML and Golgi cells, with a preference for *Gdf10*^+^ Bergmann cells in the PL compared to other variants. These results not only reveal new cell type tropisms of systemic AAVs for region-specific cell types that have not been readily accessible with IHC but also demonstrate the scalability of USeqFISH-based AAV profiling across diverse brain regions without loss of throughput or resolution.

### In situ profiling of pooled regulatory cargos

In addition to the capsid profiling, we examined the capability of USeqFISH for in situ characterization of pooled regulatory cargos of systemic AAVs. Regulatory sequences inserted in the 5′ or 3′ untranslated region (UTR) of AAV genomes have been used widely to control the expression of transgenes in targeted cell types or organs. For example, miRNA TS has been shown to have potential use for cell-type-specific transgene expression and mitigation of AAV toxicity for clinical applications owing to its ability to suppress transgene expression in the cells or tissue where the respective miRNA is highly expressed^26,70,71^. However, although sequencing studies have provided large datasets of the differential expression of miRNAs across organs and cell types, a lack of systemic approaches to validate them with AAVs has allowed us to identify only a few thus far.

**Fig. 5 |:**
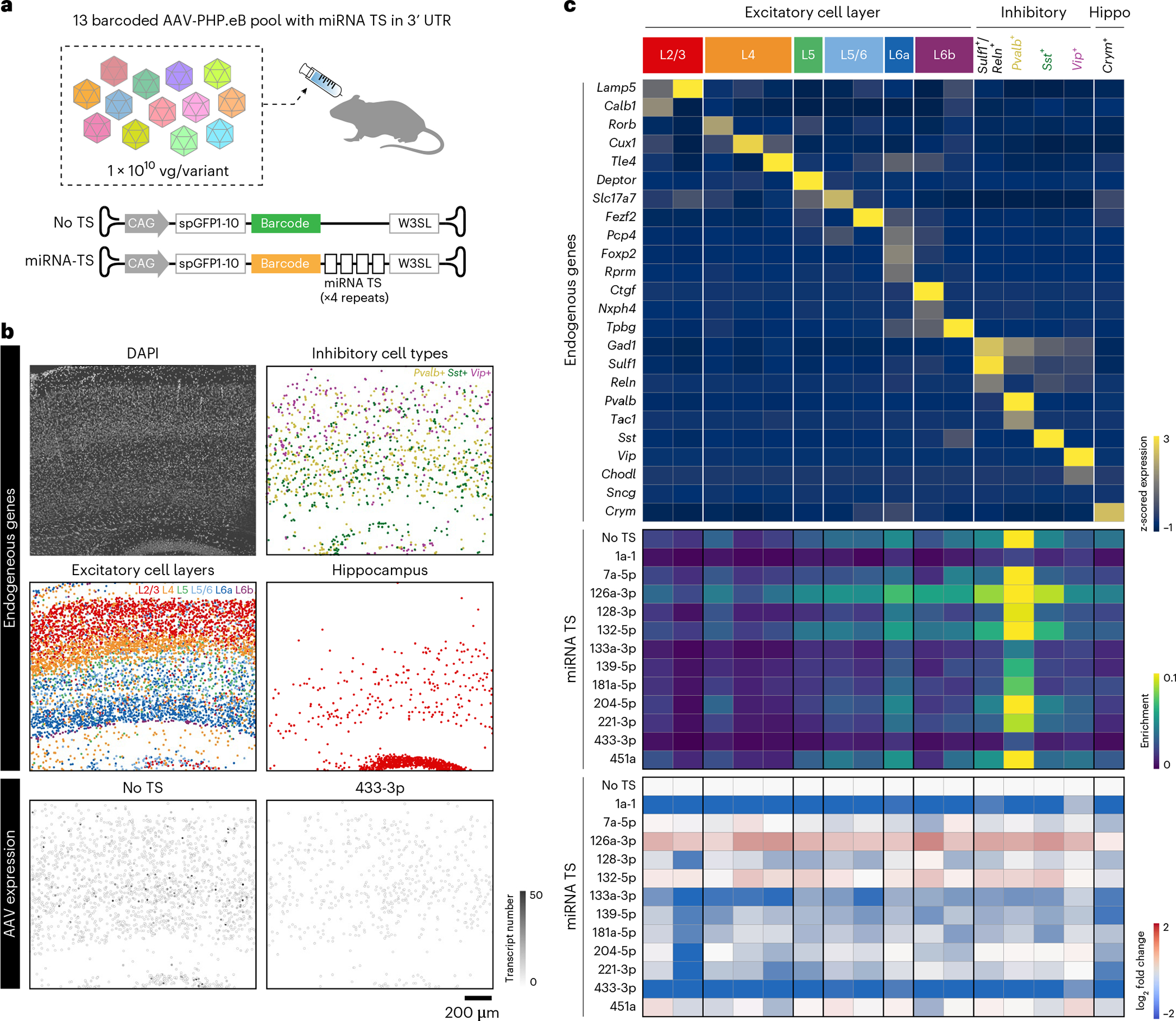
USeqFISH profiling of pooled microRNA target sites in the AAV genome across neuronal subtypes in mouse cortical layers. **a**, A pool of 13 variants (12 miRNA TSs in the 3′ UTR of the AAV genome and one control, ‘No TS’) was designed, packaged in AAV-PHP.eB and IV delivered to mice. We applied USeqFISH to the brain tissue harvested after 4 weeks of expression. **b**, Labeling of the cortex region by DAPI and the spatial location of cell types identified from clustering analysis based on endogenous gene expression. Two representative images of transgene expression (‘No TS’ and ‘433–3p’) are shown at the bottom. **c**, Endogenous (top, cividis color map) and viral gene expression profiles (enrichment: middle, viridis; log_2_ fold change: bottom, coolwarm) across the cell type clusters identified. We identified 16 total clusters, including two L2/3 (red), three L4 (orange), one L5 (green), two L5/6 (sky blue), one L6a (blue), two L6b (purple), four inhibitory and one hippocampal neuron. Colored cell types are visualized in **b**.

To test the capability of USeqFISH for high-throughput profiling of regulatory cargos, we designed a pool of 13 variants that include the four tandem repeats of miRNA TSs (a complementary sequence of microRNAs) in the 3′ UTR of AAV genomes with a unique barcode (12 variants with unique miRNA TS and one control without miRNA TS; Fig. 5a). We selected miRNA TSs, which were shown to be abundant and differently expressed across cell types, based on previous miRNA sequencing studies^72–74^. All cargos were packaged in AAV-PHP.eB, and pooled viruses were IV administered to mice at a dose of 1 × 10^10^ vg per variant (a total dose of 1.3 × 10^11^ vg per animal). After 4 weeks of expression, we harvested the brains and proceeded with USeqFISH profiling with 24 neuronal subtype marker genes (a total of 9,289 cells were analyzed in a 1.68 mm × 1.41 mm area; Fig. 5b). After the cell type clustering based on endogenous gene expression, we measured the enrichment of each variant in each cell type identified and the log_2_ fold change of enrichment compared to the control (the variant with no miRNA TS, ‘No TS’).

**Extended Data Fig. 8 |:**
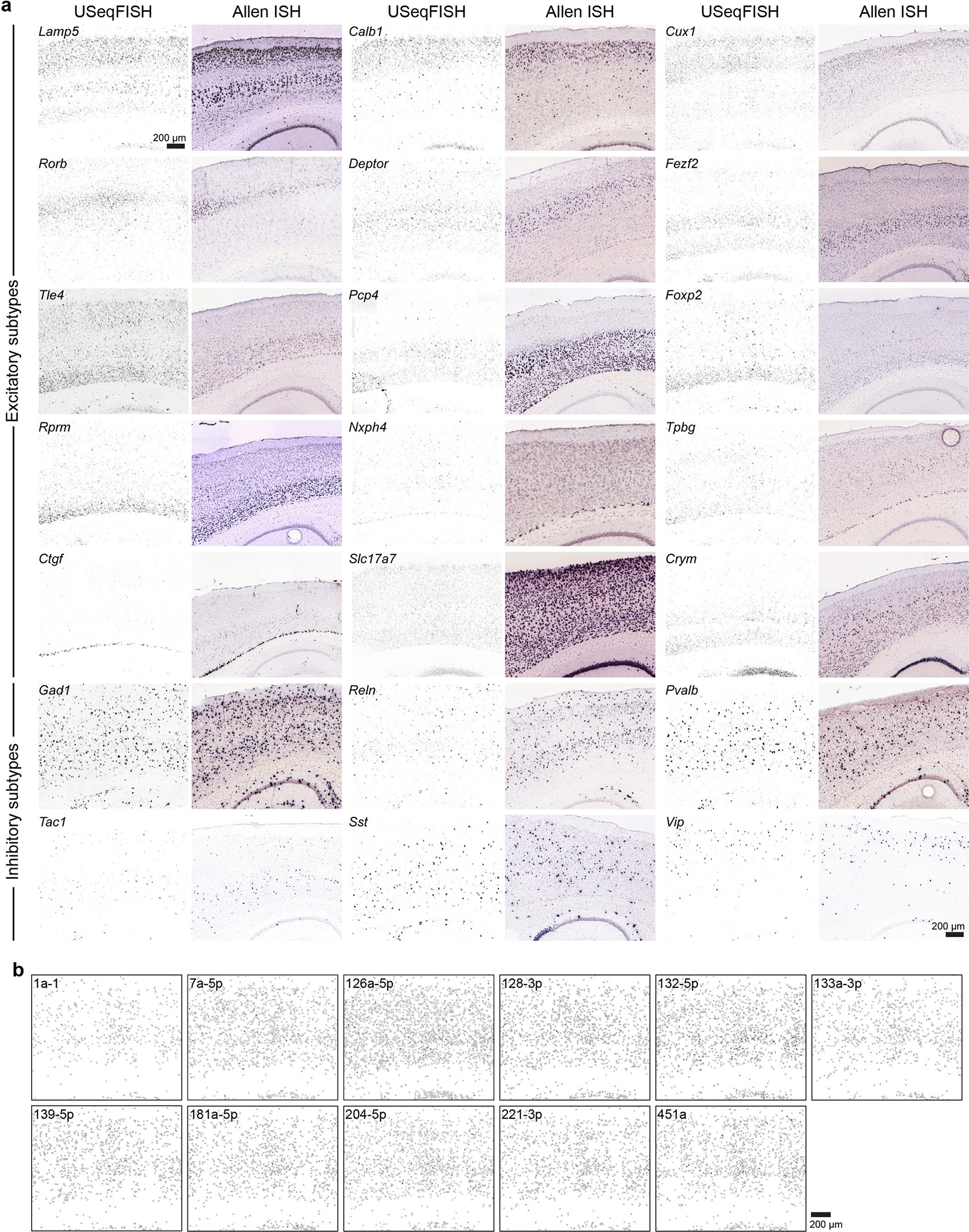
Spatial expression pattern of cell marker genes compared to Allen Brain Atlas (a) and miRNA TS pool detected by USeqFISH (b).

As a result, our selection of cell type marker genes revealed 16 clusters, including two L2/3, three L4, one L5, two L5/6, one L6a, two L6b, four inhibitory and one hippocampal neuron (Fig. 5b,c). Note that, in this experiment, we intentionally selected a different set of genes for cell type markers from the previous experiments, demonstrating the robustness of USeqFISH for cell type clustering. We also confirmed that the expression patterns of genes we selected in this experiment were consistent with the in situ hybridization data from the Allen Brain Atlas (Extended Data Fig. 8a). The overall transgene expression was enriched the most in inhibitory neurons (highest in *Pvalb*^+^ cells) and biased toward the lower layers (L5/6) of the cortex, which is consistent with our previous AAV-PHP.eB transduction profile (Fig. 4). Among these identified cell type clusters, we revealed distinct expression profiles of AAV genomes (Fig. 5c and Extended Data Fig. 8b). We found strong inhibition of transgene expression under control of miR1a-1 and miR433–3p TSs, which will potentially be useful for targeting peripheral systems with minimal toxicity to the brain. We also found that the TSs of 128–3p and 221–3p inhibit transgene expression less in inhibitory neurons than excitatory neurons, although the combination of these TSs has previously shown to have higher specificity in targeting inhibitory neurons^26^. This discrepancy could be due to differences in administration routes (systemic versus direct), the number of tandem repeats (four versus ten) and the promoter used (CAG versus hSyn). In this study, we instead found that the TS of 204–5p is promising for increasing inhibitory neuronal specificity for systemic gene delivery, because it reduces transgene expression mostly in excitatory neurons with a minimal effect on inhibitory neurons. Another interesting observation is the overall higher expression of transgenes across cell types with the TS of miR126a-3p. We speculate that the TS of miR126a-3p might reduce the endogenous level of miR126a-3p, which could increase the permeability of the BBB^75^. Although more experimental evidence will be required to understand the mechanism of miRNA TS effects on AAV transgene regulation, our results suggest that USeqFISH profiling provides an efficient approach to investigate the regulatory effect of engineered cargos for systemic AAVs in tissue.

### USeqFISH application to NHP brains

**Extended Data Fig. 9 |:**
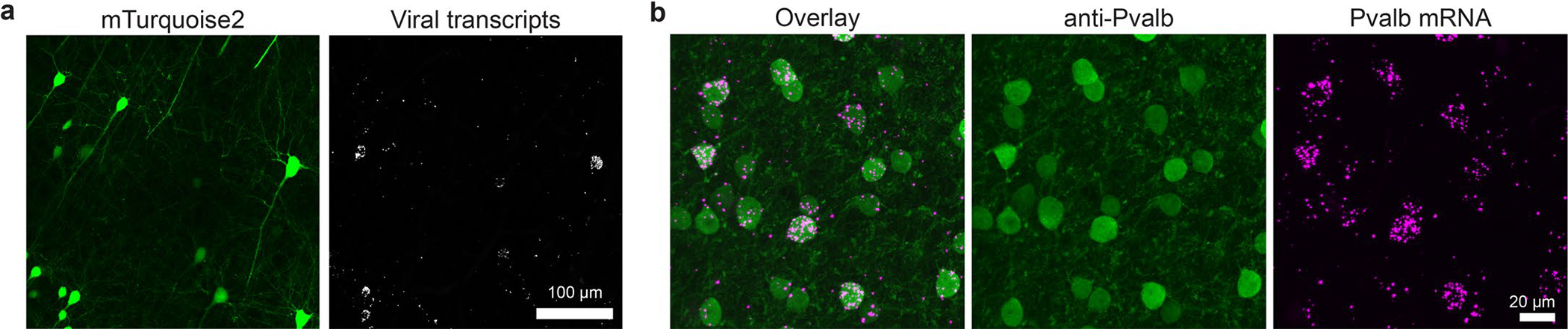
USeqFISH validation in NHP tissue. **a**, Virally labeled cells, expressing mTurquoise2 (pseudo-colored), in the rhesus macaque brain and USeqFISH labeling with probes targeting the sequence of mTurquoise2. **b**, We validated our probe design for the rhesus macaque by applying USeqFISH with *Pvalb* probes and post hoc IHC with Pvalb antibodies to the same tissue. Localization of *Pvalb* mRNA signal in cells labeled with Pvalb antibodies (the same approach used for mouse probe validation in Supplementary Fig. 3) supports the versatility of our probe design in both rodents and NHPs.

**Fig. 6 |:**
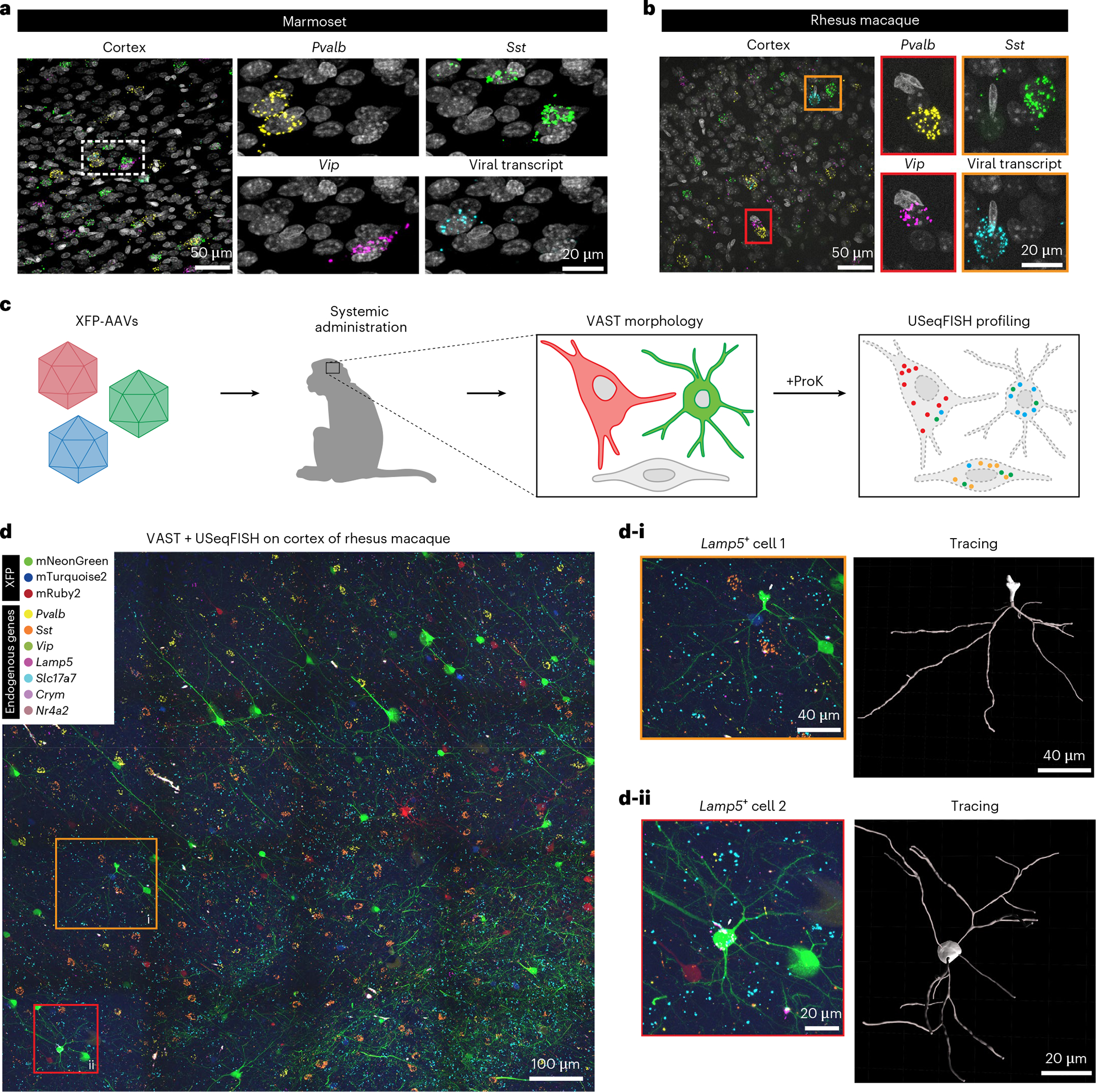
USeqFISH application to NHPs: in situ AAV detection and integrative analysis of cell morphology and transcriptional profiles. **a**,**b**, We applied USeqFISH to brain tissue slices of marmoset (**a**) and rhesus macaque (**b**) to which our viruses were administered (eight pooled variants for the marmoset and AAV.CAP-Mac for the rhesus macaque) with probes against three endogenous genes (yellow: *Pvalb*; green: *Sst*; magenta: *Vip*) and the coding sequence of each viral genome (human frataxin for the marmoset and mNeonGreen for the rhesus macaque (cyan); FPs were quenched by proteinase K (ProK) treatment). The representative images show that USeqFISH is applicable to these two NHP species with species-specific probes. **c**, Schematic of procedure of vector-assisted spectral tracing (VAST) and subsequent USeqFISH profiling of the rhesus macaque brain. We systemically delivered a cocktail of three AAV.CAP-Mac viruses packaging mNeonGreen, mTurquoise2 or mRuby2 to an infant rhesus macaque and recovered the brain. This brain exhibited a variety of colors, coming from stochastic expression of the three FPs, allowing us to trace single-cell morphologies. We additionally labeled seven endogenous genes (*Pvalb*, *Sst*, *Vip*, *Lamp5*, *Slc17a7*, *Crym* and *Nr4a2*) using USeqFISH in the same tissue to identify transcriptionally defined cell types and their morphology. **d**, Representative image of integration of VAST and USeqFISH with seven cell marker genes in the rhesus macaque brain and examples of two cells (yellow outlined box: i; red outlined box: ii) identifying both cell type and morphology.

For successful translation of engineered AAVs into therapeutic tools, they must be evaluated and characterized in NHPs, which is a considerable challenge owing to limited resources (for example, animal models, antibodies and atlases) and much longer turnaround times than in rodents. Because high-throughput, high-resolution AAV profiling could address these challenges, we sought to apply USeqFISH to NHP tissue. We reasoned that, as in mice, USeqFISH would allow us to detect synthetic transgenes, such as fluorescent proteins (FPs), in the NHP brain resulting from successful viral gene delivery. Using AAV. CAP-Mac, a capsid variant that we recently developed for efficient transduction of the rhesus macaque CNS^29^, we delivered an FP-encoding transgene and confirmed that USeqFISH with probes targeting the FP sequence can detect viral transcripts in cells expressing FPs in the NHP brain (Extended Data Fig. 9a). Detecting the endogenous RNA of NHPs requires probes specifically designed and filtered against the genes of each species. To do so, we expanded our probe design pipeline to incorporate two representative NHP species, the marmoset (*Callithrix jacchus*) and the rhesus macaque (*Macaca mulatta*), and validated the ability of USeqFISH to detect endogenous mRNAs (for example, major inhibitory subtypes: *Pvalb*, *Sst* and *Vip*) and viral transcripts expressed in the intact brain tissue of both species (Fig. 6a,b). Applying post hoc IHC to the USeqFISH-labeled NHP tissue verified the suitability of our probe design for the NHP genes and the compatibility of USeqFISH with IHC (Extended Data Fig. 9b). These results demonstrate the applicability of USeqFISH to in situ detection of endogenous and AAV-delivered genes in NHP tissues and support potential translation of USeqFISH-based AAV profiling into NHPs.

We further explored the potential application of USeqFISH, in combination with viral tools, for multimodal in situ single-cell analysis of the NHP brain. As a proof of concept, we designed an experiment to integrate viral cell morphology labeling and USeqFISH-based transcriptional profiling in the same NHP tissue. We used AAV.CAP-Mac to efficiently deliver three cargos each encoding an FP—mNG, mTurquoise2 and mRuby2—to the infant rhesus macaque via systemic administration (Fig. 6c). As a result, the brain expressed a variety of colors resulting from a stochastic mixture of the three FPs, allowing us to readily identify the morphology of single cells^29^. After imaging of FPs in the cortical area (area size: 1.14 mm × 1.14 mm × 100 μm), we treated the tissue with proteinase K to quench the FP signal and subsequently proceeded with two rounds of USeqFISH for seven cell type markers (*Pvalb*, *Sst*, *Vip*, *Lamp5*, *Slc17a7*, *Crym* and *Nr4a2*). Although coverage was sparse, this approach allowed us to trace the morphology of cells with transcriptional identities (Fig. 6d). These results demonstrate the versatility of USeqFISH and its compatibility with other single-cell labeling and barcoding methods, suggesting that it can be integrated with viral tools to explore the cellular and molecular architecture of tissue across species.

## Discussion

Fueled by directed evolution via highly multiplexed selection (for example, M-CREATE^7^, iTransduce^11^, BRAVE^30^, TRACER^12^, DELIVER^9^ and others^10,76^), AAV engineering is generating a growing number of promising vectors with potentially diversified tropism in rodents and NHPs^8^ that are awaiting thorough characterization. Here we describe a method, USeqFISH, that can fulfill this need by providing detailed transduction and tropism profiles of pooled AAVs in association with endogenous genes of infected hosts. USeqFISH profiles reveal conspicuous characteristics of each variant in the pool—for example, their enrichment in different cell types—as well as underlying tropism biases revealed by comparing variants to each other while minimizing inter-animal variability. This information predicts the performance of AAVs when singly delivered at higher dose and also identifies potential parent capsid variants that can be further evolved to improve efficiency and specificity, thereby contributing to our understanding of AAV biology and pushing forward our engineering efforts.

As a new technique for spatial transcriptomics, USeqFISH offers several improvements. First, USeqFISH is versatile, applicable to both endogenous and exogenously introduced genes in cell culture and intact tissue of mice and NHPs. Second, the USeqFISH procedure is optimized to complete rate-limiting and high-temperature steps (probe hybridization and enzyme-involving RCA) for all genes at the beginning and subsequently proceed with multiple rounds of HCR at room temperature, simplifying experimental design and instrumentation compared to other sequential labeling methods^43,44^. This indeed reduces experimental time per round between imaging, which takes ~3 hours for USeqFISH, ~5 hours for STARmap^40^ (thick tissue) and 2 days for EASI-FISH^44^, with similar scalability in thick tissue (~50 genes in USeqFISH, 28 genes in STARmap and 24 genes in EASI-FISH). Third, USeqFISH provides exceptionally bright and sensitive visualization of RNA signals with only a short unique sequence (14 nt for cell culture and 40 nt for tissue). This ability allows us to minimize the barcode and maximize the remaining space in the AAV genome, which is particularly critical for testing cargos with long promoters, tandem repeats of enhancers or microRNA target sites or even combinations of these elements. USeqFISH may also enable spatial, single-cell analysis of rare and short non-coding RNAs, which has been challenging in vivo^77^.

One caveat of USeqFISH for AAV profiling is that it can underestimate non-neuronal cells. Our computational cell segmentation based on dT labeling and the Cellpose algorithm^78^ performs better on neurons, resulting in segmentation of fewer non-neuronal cells and less accurate cell boundaries. Further optimization of cytosolic labeling and 3D segmentation algorithms for cells with various sizes and shapes would be needed to address this. Another caveat is that, although the dose used for each variant in our six-variant pool (5 × 10^10^ vg per animal) was above the detection limit that we identified from our dose study of AAV-PHP.eB, it could be too low for deep characterization of significantly less efficient variants (for example, AAV-PHP.V1 and AAV-PHP.B8). For such variants, a higher dose with a smaller pool size could be considered.

Using USeqFISH, we discovered a few features of systemic AAV capsid variants (summarized in Supplementary Fig. 6). With the ubiquitous CAG promoter, both AAV-PHP.eB and AAV.CAP-B10 efficiently transduced the mouse CNS, with the former slightly biased toward inhibitory neurons and astrocytes, and the latter exhibiting a pan-neuronal bias^8^. One particularly interesting finding was the excitatory neuronal bias of AAV-PHP.N. This variant was identified from the same 3-AA substitution library of AAV-PHP.B that yielded AAV-PHP.eB^6,7^. From sequence clustering analysis in our previous study, we identified two families sharing similar sequence motifs: AAV-PHP.eB and its relatives, sharing a similar motif close to AA588 of the AAV9 capsid sequence; and AAV-PHP.N and its relatives, sharing a 3-AA peptide close to AA589 of AAV9 (ref. ^7^). Our results suggest that this small sequence difference can alter the tropism of the AAV capsid and encourage further engineering to improve the transduction efficiency of AAV-PHP.N. We also report a new variant, AAV-PHP.AX, with a robust transduction efficiency, relatively broad coverage of neuronal subtypes and astrocytes and high tunability of cell type specificity when coupled with gene regulatory elements. In characterizing this variant, we learned that rational insertion of tropism-refining peptides into the AAV capsid sequence can alter the tropism of the capsid but not always in the way expected. These results emphasize the significance of screening-based methods for engineering capsid tropism with short mutations^30^ and using USeqFISH for AAV tropism characterization. Also, the cell type tropism biases of AAV capsids (for example, the inhibitory neuronal bias of AAV-PHP.eB, the excitatory neuronal bias of AAV-PHP.N, the broadly neuronal bias of AAV.CAP-B10 and the broadly neuronal and non-neuronal coverage of AAV-PHP.AX) suggest the potential of appropriately combining capsids and cargos with regulatory elements for targeted gene delivery with enhanced specificity.

We previously reported the successful application of droplet-based scRNA-seq for in vivo AAV transduction profiling at the transcriptomic level^32^, and USeqFISH can complement this approach. scRNA-seq provides a means to compare viral expression at greater transcriptomic level; however, it requires physical cell dissociation that can affect cellular transcription and loses spatial information. USeqFISH is limited to profiling variants within a smaller set of pre-selected endogenous genes (although this number could be expanded by adapting in situ sequencing^40,79^ or temporal barcoding^33,34,49,80^) but allows for robust spatial mapping and screening of viral expression across various regions of a tissue in a single experiment, with better sensitivity for low-abundance genes (Fig. 1k). Like the synergistic relationship of scRNA-seq and spatial transcriptomics in systems biology, we think that both can contribute to advancing AAV tools and deepening understanding of AAV biology.

USeqFISH brings spatial transcriptomics to AAV engineering, opening new directions and accelerating targeted gene delivery. We demonstrated the capability of USeqFISH for profiling systemic AAV capsids as well as regulatory cargos (miRNA TSs) in intact tissue. We anticipate that USeqFISH profiling of barcoded viral genes can be generalized to high-throughput screening of other genetic variant libraries, such as cargos with various gene regulatory elements, capsid–cargo pairs and functional readout of genetic tools, such as the CRISPR–Cas system^49^ or FLiCRE^81^, in relation to endogenous gene expression. USeqFISH can also provide a high-throughput, high-resolution AAV profiling assay for other organs or host animals with limited choices of antibodies, such as NHPs^82^. With systemic AAVs, USeqFISH would be well-suited to in vivo cellular barcoding and intact tissue profiling, which has been applied to long-range projection mapping^48^ yet remains challenging in 3D volumes. Combining USeqFISH with viral tools also offers an opportunity to integrate multimodal features, including morphology, physiology and genetics, of single cells in tissue, as we demonstrate here in NHP tissue. We believe that USeqFISH can bridge spatial transcriptomics and AAV engineering, contributing to advancing viral tools for targeted gene delivery and their broader use in basic and translational research.

### Online content

Any methods, additional references, Nature Portfolio reporting summaries, source data, extended data, supplementary information, acknowledgements, peer review information; details of author contributions and competing interests; and statements of data and code availability are available at https://doi.org/10.1038/s41587-022-01648-w.

## Methods

### Chemicals

Polyethylenimine (PEI-MAX, 24765, Polysciences), PBS (AM9625, Invitrogen), ethanol (EtOH), paraformaldehyde (PFA, RT 15714-S, Electron Microscopy Sciences), Tween 20 (P7949, MilliporeSigma), saline sodium citrate (SSC, AM9763, Invitrogen), formamide (AM9342, Invitrogen), Ribonucleoside Vanadyl Complex (RVC, S1402S, New England Biolabs (NEB)), salmon sperm DNA (15632011, Invitrogen), T4 ligase (EL0011, Thermo Fisher Scientific), BSA (B9000S, NEB), SUPERase inhibitor (AM2696, Invitrogen), Phi29 polymerase (EP0094, Thermo Fisher Scientific), dNTP (18427088, Invitrogen), 5-(3-aminoallyl)-dUTP (AM8439, Invitrogen), acrylic acid N-hydroxysuccinimide ester (AA-NHS, A8060, MilliporeSigma), acrylamide (1610140, Bio-Rad), bisacrylamide (1610142, Bio-Rad), tetramethylethylenediamine (TEMED, T7024, MilliporeSigma), ammonium persulfate (APS, A3678, MilliporeSigma), Gel Slick (50640, Lonza), HCR hairpins (Molecular Technologies), VA-044 (27776–21-2, FUJIFILM Wako Pure Chemical Corporation), sodium dodecyl sulfate (SDS, 7990-OP, Calbiochem), proteinase K (P8107S, NEB), 4′,6-diamidino-2-phenylindole (DAPI, 62248, Thermo Fisher Scientific), dimethyl sulfoxide (DMSO, D8418, MilliporeSigma), poly-l-lysine (PLL, P8920, Sigma-Aldrich), poly-d-lysine (PDL, P6407, MilliporeSigma), laminin (230017105, Thermo Fisher Scientific), ethylene carbonate (EtCB, E26258, MilliporeSigma) and dextran sulfate sodium salt (D6001, MilliporeSigma).

### Cell culture

We cultured and transfected HEK293T cells with pAAV-CMV-TET-VP3-UBC-tTA-T2A-eGFP-WPRE-hGHpA or pAAV-CAG-mNeonGreen-WPRE-hGHpA plasmid (Addgene, 99134, 100 ng ml^−1^ per well in 24-well plates) after 24–48 hours by using PEI-MAX (1:4). Three days after transfection, cells were fixed with 4% PFA for 10 minutes at room temperature and stored in 70% EtOH at −20 °C until used.

For 1-probe RCAHCR experiments, NIH3T3 cells (CRL-1658, American Type Culture Collection) were cultured in DMEM with 10% FBS. We coated glass bottom plates with 0.1 mg ml^−1^ of PDL for 1 hour at 37 °C, followed by 0.01 mg ml^−1^ of laminin for 1 hour at 37 °C. We seeded NIH3T3 cells at low density (~10% of surface area), fixed the cells in ~12 hours with 4% PFA and stored in 70% EtOH at −20 °C until used.

### Tissue slice preparation

Detailed animal procedures until tissue recovery are available below. Once harvested, the brains were sliced with a vibratome to a thickness of 50 μm (mouse and marmoset) or 75 μm (rhesus macaque). The slices were post-fixed with 4% PFA for 10 minutes at room temperature, followed by EtOH for >15 minutes at −20 °C. The slices stored in EtOH were gradually rehydrated in 75% and 50% EtOH and then washed in 1× PBS for ~30 minutes before use.

### USeqFISH protocol

For cell culture, we washed the samples with 1× PBST (0.1% Tween 20 in 1× PBS) for 1 hour and incubated with 10 nM probes in hybridization mixture (2× SSC, 10% formamide, 1% Tween 20, 20 mM RVC and 0.1 mg ml^−1^ of salmon sperm DNA) at 37 °C, overnight. Then, we washed the samples with wash buffer (2× SSC with 10% formamide) at 37 °C for 20 minutes twice and 2× SSC at 37 °C for 20 minutes twice. Next, we added the ligation mixture (T4 ligase (100 U ml^−1^) in 1× T4 ligase buffer with 1% BSA and 0.2 U μl^−1^ of SUPERase inhibitor) at room temperature, overnight. After a brief wash with 1× PBST, we added the polymerization mixture (Phi29 polymerase (200 U ml^−1^) in 1× Phi29 polymerase buffer with 1% BSA, 0.2 U μl^−1^ of SUPERase inhibitor, 250 μM dNTP and 20 μM 5-(3-aminoallyl)-dUTP) at 30 °C for 2 hours. The samples were washed with 1× PBST and then treated with AA-NHS (400 μM in 1× PBST) at room temperature for 2 hours. Next, we embedded the sample in hydrogel. The samples were immersed in hydrogel monomer solution (4% acrylamide and 0.2% bisacrylamide in 2× SSC) for 30 minutes and flattened on a glass slide. We dropped the same hydrogel solution with 0.2% TEMED and 0.2% APS to the sample and covered it with Gel Slick-coated slides. Once the gel formed in 1 hour, we detached the slide. For HCR, the initiators (10 nM in 2× SSC with 10% formamide) were added to the samples at room temperature for 30 minutes. HCR hairpins were heated at 95 °C for 90 seconds, followed by cool-down at room temperature for >30 minutes. After a brief wash with 2× SSC, hairpins (60 nM in 2× SSC) were added at room temperature for 1 hour.

For tissue slices, we added a few modifications to the protocol. First, once rehydrated, the samples were kept in the PACT monomer solution (4% acrylamide, 1% PFA and 0.25% VA-044 in 2× SSC) at 4 °C, overnight. Next, we formed the PACT gel by purging the solution with N_2_ for 5–10 minutes and immediately incubating it at 37 °C for 2 hours. After aspirating the excess gel, we washed the samples with 2× SSC 3–4 times and cleared them in 8% SDS (in 2× SSC) at 37 °C, overnight. Once cleared, the samples were washed with 2× SSC 3–4 times at room temperature for 1 day. Then, we proceeded with probe hybridization as described above. Second, before each enzyme reaction, we washed the samples with each enzyme buffer briefly. Third, for polymerization, we immersed the samples in the Phi29 polymerization mixture at 4 °C overnight before starting the reaction at 30 °C. Finally, once embedded in the hydrogel, the samples were treated with proteinase K (0.2 mg ml^−1^ in 1× PBST) at 37 °C for 1 hour before HCR amplification.

For sequential labeling, we detached hairpin assemblies and initiators from the amplicon using a two-step stripping method and added another set of initiators and hairpins for the next round. For two-step stripping, we added unique 10-nt toehold sequences to one of the hairpin pairs (Supplementary Table 2; Integrated DNA Technologies (IDT)). Each imaging round was performed as follows. We labeled the samples with DAPI (1:5,000 in 2× SSC) for 10 minutes and imaged in 2× SSC. Next, we added the displacement oligos (Supplementary Table 2; 1 μM for cell culture and 3 μM for tissue in 2× SSC) to the sample (30 minutes for cell culture and 1 hour for tissue) and, subsequently, formamide (60% in 2× SSC, 30 minutes for cell culture; 70% in 2× SSC, 1 hour for tissue) at room temperature. After washing the samples with 2× SSC, we added the initiators for the next round. Once all imaging rounds were completed, we treated the sample with DAPI for 10 minutes and dT(30) conjugated with Alexa Fluor 647 (1 μM in 2× SSC, IDT) for 1 hour for cytosolic labeling.

### Imaging

We used a Keyence fluorescence microscope (BZX-710) for cultured cells. For tissue slices, a confocal microscope (LSM 880, Zeiss, Zen for software control) with a ×10 air/×40 water immersion objective and a spinning disk confocal microscope (SDCM; Dragonfly, Andor, Fusion for software control) with a ×40/×100 oil immersion objective (Leica) and an sCMOS camera (Zyla, Andor) were used. For sequential labeling, we established an automated imaging and fluidic solution change system on the SDCM; the sample was attached to a glass coverslip pre-coated with PLL (1 mg ml^−1^) and embedded in the hydrogel. After the proteinase K treatment, the sample on the coverslip was assembled with a flow cell (FCS2, Bioptechs) connected with tubing to apply various solutions as needed at each step. Solution selection and flow control were carried out using a peristaltic pump (Minipuls 3, Gilson) and valves (MVP valves and positioners, Hamilton Company). The resolution of volume imaging was 0.151 μm per pixel in the *x* and *y* axes and 0.4–0.5 μm per pixel in the *z* axis (with a ×40 oil immersion objective). All parts of the system were automatically controlled through RS232 and REST by a custom-built Python script.

### Integration of vector-assisted spectral tracing and USeqFISH

Details of viral injection and tissue collection for the rhesus macaque are available below. After harvesting the brain, we applied USeqFISH with the same protocol except PACT clearing to the rhesus macaque brain slices expressing three FPs. Once the sample was embedded in the hydrogel for the amplicon immobilization, we set up the flow cell connected to our automated imaging and fluidics system. We first collected images of the three FPs and then flowed proteinase K (1 mg ml^−1^ in 2× SSC) through the fluidics and treated the sample for 3 hours at room temperature on the microscope. After washing with 2× SSC, we proceeded with sequential imaging for USeqFISH.

### Barcode and UGI sequence generation

We computationally generated unique barcodes and UGIs with the following criteria. We designed a random sequence (20 nt for barcodes and 19 nt for UGIs) that consisted of only three letters, A, C or T, to enhance hybridization efficiency^83^. For both barcodes and UGIs, we excluded those with more than four repeats of each letter and that had a hit against the mouse transcriptome via a BLAST search. The GC range and melting temperature (T_m_) selected for the barcode and the UGI were different (barcode: 40% ≤ GC ≤ 60%, T_m_ < 70 °C; UGI: 10% ≤ GC ≤ 20%, T_m_ < 40 °C), as were their hybridization conditions. We also performed pairwise comparisons of the new sequence with previously designed barcodes or UGIs to prevent cross-hybridization.

### Probe design for endogenous genes

To optimize probe design for USeqFISH (and HCR version 3), we improved our first version of the probe design script for HCR version 3 based on MATLAB and BLAST^84^ by importing it to Python and using Bowtie2 (ref. ^85^). This improvement made the code run much faster (<1 minute per gene) than the previous version (tens of minutes per gene). In brief, from the entire coding sequence, we selected 20-nt regions with 40% ≤ GC ≤ 60%, no more than three (for C and G) or four (for A and T) repeats, Gibb’s free energy (dG) of ≤−9 kcal per mol and unique under a Bowtie2 search. Once the target sequence candidates were identified, we aligned the whole sequence of each primer and padlock, including linkers and UGIs, with Bowtie2 again to prevent their unexpected binding to any other endogenous genes. To make the script applicable across species, we built Bowtie2 databases from GenBank genome databases: mm10 (mouse, *Mus musculus*), cj1700 (marmoset, *Callithrix jacchus*) and mmul10 (rhesus macaque, *Macaca mulatta*). All designed probes (Supplementary Table 2) were ordered through IDT and diluted in Ultrapure water before use.

### Barcoded AAV genome plasmid production

Barcoded AAV genome plasmids were based on pAAV-CAG-mNeonGreen-WPRE-hGHpA (Addgene, 99134). Double-stranded DNA (dsDNA) fragments containing the spGFP(1–10) coding sequence and the W3SL sequence, with appropriate overhangs, were synthesized as dsDNA fragments (IDT) and inserted into pAAV-CAG-mNeonGreen-WPRE-hGHpA with NEBuilder HiFi (NEB) to generate pAAV-CAG-spGFP(1–10)-W3SL. For the six-pool experiment, barcodes with 40-nt flanking sequences complementary to the acceptor vector were synthesized as dsDNA fragments (IDT). For the miRNA TS-pool experiment, AAV genomes with barcodes and miRNA TSs were generated by a commercial vendor (Alta Biotech).

### AAV production and titration

AAV production was performed using a published protocol^27^. In brief, HEK293T cells were triple transfected using PEI-MAX to deliver AAV capsid, pHelper and barcoded AAV genome plasmids. Viruses were harvested from the media and cell pellet and purified over 15%, 25%, 40% and 60% iodixanol (OptiPrep, STEMCELL Technologies) step gradients. Viruses were concentrated using Amicon Ultra centrifugal filters (MilliporeSigma), formulated in sterile PBS and titrated with qPCR by measuring the number of DNase I-resistant viral genomes relative to a linearized genome plasmid as a standard. For capsid pools, viruses were purified and titrated individually and then pooled before injection to ensure equal dosing of all variants in the pool.

For miRNA TS screening, we pooled the barcoded AAV genomes to equal concentrations before triple transfection and then titrated the entire pool with multiplexed digital droplet PCR (ddPCR). For titration, we designed sets of primers and double-quenched FAM-labeled and HEX-labeled probes (Supplementary Table 3; IDT, resuspended in pH 8 TE buffer) targeting each miRNA TS, barcode and spGFP sequence. We extracted viral genomes^27^ and performed six ten-fold serial dilutions of the extracted DNA. The final two dilutions were used for ddPCR. We loaded 3 μl of DNA into 25-μl PCR reactions (Bio-Rad, 1863024) and generated droplets from 22 μl of that PCR reaction by using droplet generation oil (Bio-Rad, 1863005) and a QX200 Droplet Generator (Bio-Rad). After transferring 40 μl of droplets to a 96-well PCR plate and sealing the plate with a pierceable heat seal (Bio-Rad, 1814040 and 1814000), we ran the PCR according to the manufacturerʼs protocol. After PCR, we measured droplets with a QX200 Droplet Reader and analyzed the data with the QX Manager software (Bio-Rad, 12010213). Within each well, the concentrations of one specific genome variant (miRNA TS and barcode) and all AAV genomes (spGFP) were measured to calculate the ratio of [genome variant] to [total genome], and the mean was used for normalization.

### Virus injection and tissue recovery

For mice, all animal procedures were approved by the California Institute of Technology (Caltech) Institutional Animal Care and Use Committee (IACUC). We administered AAVs via retro-orbital injection during anesthesia with isoflurane (1–3% in 95% O_2_/5% CO_2_ provided via a nose cone at 1 L min^−1^) and, subsequently, 1–2 drops of 0.5% proparacaine to the corneal surface^27^. After 3–4 weeks of expression, the animals were sacrificed by transcardiac perfusion with 1× PBS, followed by 4% PFA. The brain was harvested and post-fixed in 4% PFA at 4 °C for overnight.

For the marmoset, all animal procedures were approved by the University of California, San Diego IACUC, and details are available in ref. ^82^. Ketamine (20 mg kg^−1^) and acepromazine (0.5 mg kg^−1^) were intramuscularly injected to anesthetize the animal. An IV catheter was placed in the saphenous vein of the hind leg and sequentially flushed with ~2 ml of lactated Ringer’s solution (LRS) for 2 minutes, pooled virus (~500–900 μl; 200 μl min^−1^; eight capsid variants carrying the same coding sequence, human frataxin, with an HA tag) and ~3 ml of LRS. After 6 weeks of expression, the animal was euthanized by intraperitoneal injection of pentobarbital. The brain was harvested, flash-frozen in 2-methylbutane chilled with dry ice and stored in −80 °C until used.

For the rhesus macaque, all animal procedures were approved by the IACUC at the University of California, Davis, and the California National Primate Research Center (CNPRC), and details are available in ref. ^29^. One newborn animal anesthetized with ketamine (0.1 ml) was IV infused with a cocktail of three AAV.CAP-Mac^29^ vectors carrying mNeonGreen, mTurquoise2 or mRuby2, respectively, under control of the CAG promoter into the saphenous vein (<750 μl for ~1 minute). Eleven weeks after injection, the animal was anesthetized with sodium pentobarbital in accordance with guidelines for humane euthanasia of animals at the CNPRC. After being perfused with 1× PBS and 4% PFA, the brain was harvested and sectioned into 4-mm coronal blocks. All tissue was post-fixed in 4% PFA for 3 days and then transferred to Caltech for further processing.

### Immunohistochemistry

The mouse brain slices were incubated in blocking buffer (1× PBS with 10% donkey serum and 1% BSA) with primary antibodies (Aves GFP-1020, 1:1,000; anti-GFAP, 829401, BioLegend, 1:1,000; anti-Glut1, 07–1401, MilliporeSigma, 1:400; anti-actin, α-smooth muscle-cy3, C6198, MilliporeSigma, 1:1,000) at room temperature overnight. After being washed twice with 1× PBS for 30 minutes, the samples were incubated in blocking buffer with secondary antibodies (goat anti-chicken IgY, Alexa Fluor 633, A21103, Invitrogen, 1:1,000; goat anti-rabbit IgG, Alexa Fluor 633, A21070, Invitrogen, 1:1,000) for 1 hour at room temperature.

For the rhesus macaque brain slices, we labeled the primary antibody (anti-Pvalb, ab181086, Abcam, 1:200) in buffer (1× PBS with 10% donkey serum and 0.1% Triton X-100) at room temperature overnight, washed the samples with 1× PBS for 10 minutes, three times, and then labeled the secondary antibody (donkey anti-rabbit IgG, Jackson ImmunoResearch, 1:200) in the same buffer at room temperature overnight. For the last step of both samples, we briefly washed them with 1× PBS and mounted the samples on glass slides with Prolong Diamond Antifade Mountant (P36970, Molecular Probes).

### Fluorescence in situ hybridization (HCR and smFISH)

We designed probes for HCR v3 (ref. ^54^) (Supplementary Table 2) using our custom script and synthesized through IDT. We first incubated the samples in hybridization buffer (2 nM probes in 2× SSC with 10% EtCB and 10% dextran sulfate) at 37 °C overnight. We then washed the samples with stringent wash buffer (10% EtCB in 2× SSC) at 37 °C for 30 minutes, twice, followed by additional wash with 2× SSC at room temperature for 30 minutes, twice. For amplification, HCR hairpins were heated at 95 °C for 90 seconds and cooled down to room temperature for >30 minues. The samples were incubated in amplification buffer (60 nM hairpins in 2× SSC with 10% dextran sulfate) at room temperature overnight. To achieve single-molecular resolution HCR (smHCR), the amplification was performed for 1 hour^54^.

For smFISH, we used commercial probes (Stellaris FISH Probes, Mouse Gapdh with Quasar 570 Dye, SMF-3002–1; Stellaris FISH Probes, Mouse Tfrc with Quasar 570 Dye, SMF-3007–1; Stellaris FISH Probes, Mouse Polr2a with Quasar 570 Dye, SMF-3005–1) and buffers (Stellaris RNA FISH Hybridization Buffer, SMF-HB1–10; Stellaris RNA FISH Wash Buffer A, SMF-WA1–60; Stellaris RNA FISH Wash Buffer B, SMF-WB1–20), following the ‘Adherent cells’ protocol.

### Data analysis

For USeqFISH performance characterization (Fig. 1) and the dose-dependency experiment (Fig. 3), we used a maximum intensity projection of the ~20-μm-thick volume. Quantification of the RNA signal intensity was performed as follows. We subtracted the background calculated by applying the area_opening function in scikit-image. After removing small objects with double erosion, we identified the foreground pixels and measured the mean intensity of the background to calculate the cumulative histogram of the signal intensity and the SBR (the intensity of the foreground pixels / the mean value of the background intensity). For quantifying RNA spots, we created a mask of the DAPI signal manually using Fiji and processed other channels with RNA spots as follows. We subtracted the background as described above and applied the Laplacian of Gaussian filter to detect RNA spots. Then, we calculated the distance of each spot to all nuclei and assigned it to the closest nucleus only if the distance was <10 μm.

For the pool studies (Figs. 3–5), we developed a computational analysis pipeline that includes registration, spot detection and cell segmentation in a 3D volume (Extended Data Fig. 6a). First, we exploited Cellpose^78^ with the dT(30)-labeled image to segment single-cell bodies in the 3D volume. We found that downsampling the dT-labeled image to make each cell have an estimated diameter of ~30 pixels worked quickly and produced the best result in single-cell segmentation. With the labeled mask of each cell, we performed a convex hull operation to smooth cell boundaries. Second, we identified the RNA spots of each channel in each round by applying the Laplacian of a Gaussian filter. Third, we acquired the transformation matrix by using phase cross-correlation of the DAPI image at each round to the last DAPI image. We added this transformation matrix to the one to correct optical aberration that we obtained with fluorescence microbeads (FocalCheck Fluorescence Microscope Test slides #1, F36909, Thermo Fisher Scientific) before the experiment. Finally, we combined all three pieces of information (segmented cells, detected spots and registration coordinates) to assign the spots to individual cells and finally obtained the expression matrix of endogenous and viral genes in each cell. For tiled datasets (cortex layers; Figs. 4a and 5b), we processed all individual tiles to get the expression matrices of each, and cells in the overlap between tiles (10%) were excluded from one tile for the clustering analysis below. Tiled images were stitched in Fusion (Andor) for visualization. The pipeline was parallelized using Dask to accelerate the processing. The total processing time was 10–20 minutes for Cellpose cell segmentation (with GPU) and 10–20 minutes per round (for registration and spot detection of all four channels) on clusters at the Caltech Resnick High Performance Computing Center.

The quantitative analysis of the expression matrix was conducted mainly with Scanpy^86^ (Extended Data Fig. 6b) on a standalone laptop. In brief, we used only the endogenous gene expression matrix of all cells to identify cell types as follows. Based on the distribution of total spot counts, we filtered cells with no RNA spots or too many (usually <5 cells from the entire dataset). After normalization and z-standardization of the data, we applied principal component analysis and Leiden clustering to the data to identify cell type clusters. We performed subclustering with the large clusters and merged the clusters based on Ward distance until the elbow point. Once the type of each cell was determined, we calculated (1) enrichment by calculating mean of log-transformed (log1p) spot counts per cell and (2) relative tropism bias by calculating mean of log-normalized and z-scored spot counts per cell. For additional information, we provide (1) transduction efficiency measured by dividing the number of cells having one or more of each viral barcode by the total cell number in each cluster and (2) mean spot numbers per cell measured by averaging the spot numbers of each virus in transduced cells in each cluster for all data in Supplementary Fig. 6. Images were visualized using Napari, Fiji or Imaris 9.5 for 3D views.

### Statistics and reproducibility

All in vitro experiments were repeated at least three times with similar results. All in vivo experiments with mice were repeated at least twice using 2–5 animals with similar results. All NHP experiments were repeated at least twice using one animal with similar results.

## Extended Data

## Supplementary Material

Table 3

Table 1

Table 2

Supplementary Info

## Data Availability

All sequences of probes and primers used in this study are provided in Supplementary Tables 2 and 3. We used GenBank genome assemblies (mm10 for mouse, cj1700 for marmoset and mmul10 for rhesus macaque) to build Bowtie2 databases for probe design. We used previously published data^65^ (Gene Expression Omnibus, GSE71585) in Supplementary Fig. 5b. The vector plasmid used to produce AAV-PHP.AX is available at Addgene (195218). Raw image datasets for pooled screening experiments are deposited in the Brain Image Library (https://doi.org/10.35077/g.529). Other data that support the findings of this study are available from the corresponding author upon reasonable request.

## References

[1] Nectow AR & Nestler EJ Viral tools for neuroscience. Nat. Rev. Neurosci. 21, 669–681 (2020). 33110222 10.1038/s41583-020-00382-z PMC7808553

[2] Bedbrook CN, Deverman BE & Gradinaru V Viral strategies for targeting the central and peripheral nervous systems. Annu. Rev. Neurosci. 41, 323–348 (2018). 29709207 10.1146/annurev-neuro-080317-062048

[3] Zhu D, Schieferecke AJ, Lopez PA & Schaffer DV Adeno-associated virus vector for central nervous system gene therapy. Trends Mol. Med. 27, 524–537 (2021). 33895085 10.1016/j.molmed.2021.03.010

[4] Wang D, Tai PWL & Gao G Adeno-associated virus vector as a platform for gene therapy delivery. Nat. Rev. Drug Discov. 18, 358–378 (2019). 30710128 10.1038/s41573-019-0012-9 PMC6927556

[5] Deverman BE Cre-dependent selection yields AAV variants for widespread gene transfer to the adult brain. Nat. Biotechnol. 34, 204–209 (2016). 26829320 10.1038/nbt.3440 PMC5088052

[6] Chan KY Engineered AAVs for efficient noninvasive gene delivery to the central and peripheral nervous systems. Nat. Neurosci. 20, 1172–1179 (2017). 28671695 10.1038/nn.4593 PMC5529245

[7] Ravindra Kumar S Multiplexed Cre-dependent selection yields systemic AAVs for targeting distinct brain cell types. Nat. Methods 17, 541–550 (2020). 32313222 10.1038/s41592-020-0799-7 PMC7219404

[8] Goertsen D AAV capsid variants with brain-wide transgene expression and decreased liver targeting after intravenous delivery in mouse and marmoset. Nat. Neurosci. 25, 106–115 (2021). 34887588 10.1038/s41593-021-00969-4

[9] Tabebordbar M Directed evolution of a family of AAV capsid variants enabling potent muscle-directed gene delivery across species. Cell 184, 4919–4938 (2021). 34506722 10.1016/j.cell.2021.08.028 PMC9344975

[10] Ojala DS In vivo selection of a computationally designed SCHEMA AAV library yields a novel variant for infection of adult neural stem cells in the SVZ. Mol. Ther. 26, 304–319 (2018). 28988711 10.1016/j.ymthe.2017.09.006 PMC5762983

[11] Hanlon KS Selection of an efficient AAV vector for robust CNS transgene expression. Mol. Ther. Methods Clin. Dev. 15, 320–332 (2019). 31788496 10.1016/j.omtm.2019.10.007 PMC6881693

[12] Nonnenmacher M Rapid evolution of blood-brain-barrier-penetrating AAV capsids by RNA-driven biopanning. Mol. Ther. Methods Clin. Dev. 20, 366–378 (2021). 33553485 10.1016/j.omtm.2020.12.006 PMC7841218

[13] Weinmann J Identification of a myotropic AAV by massively parallel in vivo evaluation of barcoded capsid variants. Nat. Commun. 11, 5432 (2020). 33116134 10.1038/s41467-020-19230-w PMC7595228

[14] de Leeuw CN Targeted CNS delivery using human MiniPromoters and demonstrated compatibility with adeno-associated viral vectors. Mol. Ther. Methods Clin. Dev. 1, 5 (2014). 10.1038/mtm.2013.5 PMC3992516 24761428

[15] Haery L Adeno-associated virus technologies and methods for targeted neuronal manipulation. Front. Neuroanat. 13, 93 (2019). 31849618 10.3389/fnana.2019.00093 PMC6902037

[16] Nitta K, Matsuzaki Y, Konno A & Hirai H Minimal purkinje cell-specific PCP2/L7 promoter virally available for rodents and non-human primates. Mol. Ther. Methods Clin. Dev. 6, 159–170 (2017). 28828391 10.1016/j.omtm.2017.07.006 PMC5552061

[17] Hoshino C GABAergic neuron-specific whole-brain transduction by AAV-PHP.B incorporated with a new GAD65 promoter. Mol. Brain 14, 33 (2021). 33588899 10.1186/s13041-021-00746-1 PMC7885384

[18] Mehta P Functional access to neuron subclasses in rodent and primate forebrain. Cell Rep. 26, 2818–2832 (2019). 30840900 10.1016/j.celrep.2019.02.011 PMC6509701

[19] Graybuck LT Enhancer viruses for combinatorial cell-subclass-specific labeling. Neuron 109, 1449–1464 (2021). 33789083 10.1016/j.neuron.2021.03.011 PMC8610077

[20] Hrvatin S A scalable platform for the development of cell-type-specific viral drivers. eLife 8, e48089 (2019). 31545165 10.7554/eLife.48089 PMC6776442

[21] Mich JK Functional enhancer elements drive subclass-selective expression from mouse to primate neocortex. Cell Rep. 34, 108754 (2021). 33789096 10.1016/j.celrep.2021.108754 PMC8163032

[22] Rubin AN Regulatory elements inserted into AAVs confer preferential activity in cortical interneurons. eNeuro 7, ENEURO.0211–20.2020 (2020). 10.1523/ENEURO.0211-20.2020 PMC7768279 33199411

[23] Vormstein-Schneider D Viral manipulation of functionally distinct interneurons in mice, non-human primates and humans. Nat. Neurosci. 23, 1629–1636 (2020). 32807948 10.1038/s41593-020-0692-9 PMC8015416

[24] Dimidschstein J A viral strategy for targeting and manipulating interneurons across vertebrate species. Nat. Neurosci. 19, 1743–1749 (2016). 27798629 10.1038/nn.4430 PMC5348112

[25] Nair RR, Blankvoort S, Lagartos MJ & Kentros C Enhancer-Driven Gene Expression (EDGE) enables the generation of viral vectors specific to neuronal subtypes. iScience 23, 100888 (2020). 32087575 10.1016/j.isci.2020.100888 PMC7033522

[26] Keaveney MK A microRNA-based gene-targeting tool for virally labeling interneurons in the rodent cortex. Cell Rep. 24, 294–303 (2018). 29996091 10.1016/j.celrep.2018.06.049 PMC6095194

[27] Challis RC Systemic AAV vectors for widespread and targeted gene delivery in rodents. Nat. Protoc. 14, 379–414 (2019). 30626963 10.1038/s41596-018-0097-3 PMC13333184

[28] Xie J MicroRNA-regulated, systemically delivered rAAV9: a step closer to CNS-restricted transgene expression. Mol. Ther. 19, 526–535 (2011). 21179009 10.1038/mt.2010.279 PMC3048189

[29] Chuapoco MR Intravenous gene transfer throughout the brain of infant Old World primates using AAV. Preprint at 10.1101/2022.01.08.475342v1 (2022).

[30] Davidsson M A systematic capsid evolution approach performed in vivo for the design of AAV vectors with tailored properties and tropism. Proc. Natl Acad. Sci. USA 116, 27053–27062 (2019). 31818949 10.1073/pnas.1910061116 PMC6936499

[31] Kondratov O A comprehensive study of a 29-capsid AAV library in a non-human primate central nervous system. Mol. Ther. J. Am. Soc. Gene Ther. 29, 2806–2820 (2021). 10.1016/j.ymthe.2021.07.010 PMC8417503 34298128

[32] Brown D Deep parallel characterization of AAV tropism and AAV-mediated transcriptional changes via single-cell RNA sequencing. Front. Immunol. 12, 4117 (2021). 10.3389/fimmu.2021.730825 PMC8574206 34759919

[33] Eng C-HL Transcriptome-scale super-resolved imaging in tissues by RNA seqFISH+. Nature 568, 235–239 (2019). 30911168 10.1038/s41586-019-1049-y PMC6544023

[34] Chen KH, Boettiger AN, Moffitt JR, Wang S & Zhuang X Spatially resolved, highly multiplexed RNA profiling in single cells. Science 348, aaa6090 (2015). 25858977 10.1126/science.aaa6090 PMC4662681

[35] Rodriques SG Slide-seq: a scalable technology for measuring genome-wide expression at high spatial resolution. Science 363, 1463–1467 (2019). 30923225 10.1126/science.aaw1219 PMC6927209

[36] Lee JH Highly multiplexed subcellular RNA sequencing in situ. Science 343, 1360–1363 (2014). 24578530 10.1126/science.1250212 PMC4140943

[37] Cho C-S Microscopic examination of spatial transcriptome using Seq-Scope. Cell 184, 3559–3572 (2021). 34115981 10.1016/j.cell.2021.05.010 PMC8238917

[38] Ortiz C, Carlén M & Meletis K Spatial transcriptomics: molecular maps of the mammalian brain. Annu. Rev. Neurosci. 44, 547–562 (2021). 33914592 10.1146/annurev-neuro-100520-082639

[39] Rao A, Barkley D, França GS & Yanai I Exploring tissue architecture using spatial transcriptomics. Nature 596, 211–220 (2021). 34381231 10.1038/s41586-021-03634-9 PMC8475179

[40] Wang X Three-dimensional intact-tissue sequencing of single-cell transcriptional states. Science 361, eaat5691 (2018). 29930089 10.1126/science.aat5691 PMC6339868

[41] Chen F Nanoscale imaging of RNA with expansion microscopy. Nat. Methods 13, 679–684 (2016). 27376770 10.1038/nmeth.3899 PMC4965288

[42] Alon S Expansion sequencing: spatially precise in situ transcriptomics in intact biological systems. Science 371, eaax2656 (2021). 33509999 10.1126/science.aax2656 PMC7900882

[43] Codeluppi S Spatial organization of the somatosensory cortex revealed by osmFISH. Nat. Methods 15, 932–935 (2018). 30377364 10.1038/s41592-018-0175-z

[44] Wang Y EASI-FISH for thick tissue defines lateral hypothalamus spatio-molecular organization. Cell 184, 6361–6377 (2021). 34875226 10.1016/j.cell.2021.11.024

[45] Gyllborg D Hybridization-based in situ sequencing (HybISS) for spatially resolved transcriptomics in human and mouse brain tissue. Nucleic Acids Res. 48, e112 (2020). 32990747 10.1093/nar/gkaa792 PMC7641728

[46] Emanuel G, Moffitt JR & Zhuang X High-throughput, image-based screening of pooled genetic-variant libraries. Nat. Methods 14, 1159–1162 (2017). 29083401 10.1038/nmeth.4495 PMC5958624

[47] Feldman D Optical pooled screens in human cells. Cell 179, 787–799 (2019). 31626775 10.1016/j.cell.2019.09.016 PMC6886477

[48] Sun Y-C Integrating barcoded neuroanatomy with spatial transcriptional profiling enables identification of gene correlates of projections. Nat. Neurosci. 24, 873–885 (2021). 33972801 10.1038/s41593-021-00842-4 PMC8178227

[49] Askary A In situ readout of DNA barcodes and single base edits facilitated by in vitro transcription. Nat. Biotechnol. 38, 66–75 (2020). 31740838 10.1038/s41587-019-0299-4 PMC6954335

[50] Chow K-HK Imaging cell lineage with a synthetic digital recording system. Science 372, eabb30990 (2021). 10.1126/science.abb3099 33833095

[51] Wang SK, Lapan SW, Hong CM, Krause TB & Cepko CL In situ detection of adeno-associated viral vector genomes with SABER-FISH. Mol. Ther. Methods Clin. Dev. 19, 376–386 (2020). 33209963 10.1016/j.omtm.2020.10.003 PMC7658570

[52] Zhao J High-resolution histological landscape of AAV DNA distribution in cellular compartments and tissues following local and systemic injection. Mol. Ther. Methods Clin. Dev. 18, 856–868 (2020). 32953935 10.1016/j.omtm.2020.08.006 PMC7479330

[53] Larsson C, Grundberg I, Söderberg O & Nilsson M In situ detection and genotyping of individual mRNA molecules. Nat. Methods 7, 395–397 (2010). 20383134 10.1038/nmeth.1448

[54] Choi HMT Third-generation in situ hybridization chain reaction: multiplexed, quantitative, sensitive, versatile, robust. Development 145, dev165753 (2018). 29945988 10.1242/dev.165753 PMC6031405

[55] Terashima T Gene therapy for neuropathic pain through siRNA-IRF5 gene delivery with homing peptides to microglia. Mol. Ther. Nucleic Acids 11, 203–215 (2018). 29858055 10.1016/j.omtn.2018.02.007 PMC5992689

[56] Raj A, van den Bogaard P, Rifkin SA, van Oudenaarden A & Tyagi S Imaging individual mRNA molecules using multiple singly labeled probes. Nat. Methods 5, 877–879 (2008). 18806792 10.1038/nmeth.1253 PMC3126653

[57] Kishi JY SABER amplifies FISH: enhanced multiplexed imaging of RNA and DNA in cells and tissues. Nat. Methods 16, 533–544 (2019). 31110282 10.1038/s41592-019-0404-0 PMC6544483

[58] Shah S Single-molecule RNA detection at depth by hybridization chain reaction and tissue hydrogel embedding and clearing. Development 143, 2862–2867 (2016). 27342713 10.1242/dev.138560 PMC5004914

[59] Yang B Single-cell phenotyping within transparent intact tissue through whole-body clearing. Cell 158, 945–958 (2014). 25088144 10.1016/j.cell.2014.07.017 PMC4153367

[60] Treweek JB Whole-body tissue stabilization and selective extractions via tissue-hydrogel hybrids for high-resolution intact circuit mapping and phenotyping. Nat. Protoc. 10, 1860–1896 (2015). 26492141 10.1038/nprot.2015.122 PMC4917295

[61] Shah S, Lubeck E, Zhou W & Cai L In situ transcription profiling of single cells reveals spatial organization of cells in the mouse hippocampus. Neuron 92, 342–357 (2016). 27764670 10.1016/j.neuron.2016.10.001 PMC5087994

[62] Choi J-H Optimization of AAV expression cassettes to improve packaging capacity and transgene expression in neurons. Mol. Brain 7, 17 (2014). 24618276 10.1186/1756-6606-7-17 PMC3975461

[63] Cabantous S, Terwilliger TC & Waldo GS Protein tagging and detection with engineered self-assembling fragments of green fluorescent protein. Nat. Biotechnol. 23, 102–107 (2005). 15580262 10.1038/nbt1044

[64] Tasic B Shared and distinct transcriptomic cell types across neocortical areas. Nature 563, 72–78 (2018). 30382198 10.1038/s41586-018-0654-5 PMC6456269

[65] Tasic B Adult mouse cortical cell taxonomy revealed by single cell transcriptomics. Nat. Neurosci. 19, 335–346 (2016). 26727548 10.1038/nn.4216 PMC4985242

[66] Gokce O Cellular taxonomy of the mouse striatum as revealed by single-cell RNA-seq. Cell Rep. 16, 1126–1137 (2016). 27425622 10.1016/j.celrep.2016.06.059 PMC5004635

[67] Kozareva V A transcriptomic atlas of mouse cerebellar cortex comprehensively defines cell types. Nature 598, 214–219 (2021). 34616064 10.1038/s41586-021-03220-z PMC8494635

[68] Phillips JW A repeated molecular architecture across thalamic pathways. Nat. Neurosci. 22, 1925–1935 (2019). 31527803 10.1038/s41593-019-0483-3 PMC6819258

[69] Nathanson JL, Yanagawa Y, Obata K & Callaway EM Preferential labeling of inhibitory and excitatory cortical neurons by endogenous tropism of adeno-associated virus and lentivirus vectors. Neuroscience 161, 441–450 (2009). 19318117 10.1016/j.neuroscience.2009.03.032 PMC2728494

[70] Qiao C Liver-specific microRNA-122 target sequences incorporated in AAV vectors efficiently inhibits transgene expression in the liver. Gene Ther. 18, 403–410 (2011). 21150938 10.1038/gt.2010.157 PMC3686499

[71] Hordeaux J MicroRNA-mediated inhibition of transgene expression reduces dorsal root ganglion toxicity by AAV vectors in primates. Sci. Transl. Med. 12, eaba9188 (2020). 33177182 10.1126/scitranslmed.aba9188

[72] Pena JTG miRNA in situ hybridization in formaldehyde and EDC-fixed tissues. Nat. Methods 6, 139–141 (2009). 19137005 10.1038/nmeth.1294 PMC2838186

[73] He M Cell-type-based analysis of microRNA profiles in the mouse brain. Neuron 73, 35–48 (2012). 22243745 10.1016/j.neuron.2011.11.010 PMC3270494

[74] Isakova A, Fehlmann T, Keller A & Quake SR A mouse tissue atlas of small noncoding RNA. Proc. Natl Acad. Sci. USA 117, 25634–25645 (2020). 32978296 10.1073/pnas.2002277117 PMC7568261

[75] Fu X, Niu T & Li X MicroRNA-126–3p attenuates intracerebral hemorrhage-induced blood–brain barrier disruption by regulating VCAM-1 expression. Front. Neurosci. 13, 866 (2019). 31474826 10.3389/fnins.2019.00866 PMC6707088

[76] Tervo DGR A designer AAV variant permits efficient retrograde access to projection neurons. Neuron 92, 372–382 (2016). 27720486 10.1016/j.neuron.2016.09.021 PMC5872824

[77] Liu S Barcoded oligonucleotides ligated on RNA amplified for multiplexed and parallel in situ analyses. Nucleic Acids Res. 49, e58 (2021). 33693773 10.1093/nar/gkab120 PMC8191787

[78] Stringer C, Wang T, Michaelos M & Pachitariu M Cellpose: a generalist algorithm for cellular segmentation. Nat. Methods 18, 100–106 (2021). 33318659 10.1038/s41592-020-01018-x

[79] Lee JH Fluorescent in situ sequencing (FISSEQ) of RNA for gene expression profiling in intact cells and tissues. Nat. Protoc. 10, 442–458 (2015). 25675209 10.1038/nprot.2014.191 PMC4327781

[80] Lubeck E, Coskun AF, Zhiyentayev T, Ahmad M & Cai L Single-cell in situ RNA profiling by sequential hybridization. Nat. Methods 11, 360–361 (2014). 24681720 10.1038/nmeth.2892 PMC4085791

[81] Kim CK A molecular calcium integrator reveals a striatal cell type driving aversion. Cell 183, 2003–2019 (2020). 33308478 10.1016/j.cell.2020.11.015 PMC9839359

[82] Chen X Engineered AAVs for non-invasive gene delivery to rodent and non-human primate nervous systems. Neuron 110, 2242–2257 (2022). 35643078 10.1016/j.neuron.2022.05.003 PMC9308721

[83] Moffitt JR High-throughput single-cell gene-expression profiling with multiplexed error-robust fluorescence in situ hybridization. Proc. Natl Acad. Sci. USA 113, 11046–11051 (2016). 27625426 10.1073/pnas.1612826113 PMC5047202

[84] Patriarchi T Ultrafast neuronal imaging of dopamine dynamics with designed genetically encoded sensors. Science. 360, eaat4422 (2018). 29853555 10.1126/science.aat4422 PMC6287765

[85] Langmead B & Salzberg SL Fast gapped-read alignment with Bowtie 2. Nat. Methods 9, 357–359 (2012). 22388286 10.1038/nmeth.1923 PMC3322381

[86] Wolf FA, Angerer P & Theis FJ SCANPY: large-scale single-cell gene expression data analysis. Genome Biol. 19, 15 (2018). 29409532 10.1186/s13059-017-1382-0 PMC5802054

[87] Jang MJ Spatial transcriptomics for profiling the tropism of viral vectors in tissues. https://github.com/GradinaruLab/useqfish_probedesign (2022). 10.1038/s41587-022-01648-w PMC10443732 36702899

[88] Jang MJ Spatial transcriptomics for profiling the tropism of viral vectors in tissues. https://github.com/GradinaruLab/useqfish_imaging (2022). 10.1038/s41587-022-01648-w PMC10443732 36702899

[89] Jang MJ Spatial transcriptomics for profiling the tropism of viral vectors in tissues. https://github.com/GradinaruLab/useqfish_analysis (2022). 10.1038/s41587-022-01648-w PMC10443732 36702899

