# Supplementary material for: Spatial transcriptomics for profiling the tropism of viral vectors in tissues
Source: Nat Biotechnol. 2023 Jan 26;41(9):1272–86. doi: 10.1038/s41587-022-01648-w (PMC10443732; doi:10.1038/s41587-022-01648-w)
Supplement: Supplementary file 1 — Supplementary Figs. 1–6 [file 41587_2022_1648_MOESM1_ESM.pdf]

nature biotechnology

Article

https://doi.org/10.1038/s41587-022-01648-w

Spatial transcriptomics for profiling the tropism of viral vectors in tissues

In the format provided by the authors and unedited

Supplementary Information

Table of contents

Supplementary Figure 1. Optimization of RNA-retained PACT
Supplementary Figure 2. Position and detection of barcodes inserted in the viral genome
Supplementary Figure 3. Probe design validation with post-hoc immunohistochemistry or transgenic reporter mouse lines
Supplementary Figure 4. Comparison of major cell type tropism profiling between two mice
Supplementary Figure 5. USeqFISH detection of endogenous genes in mouse cortex volume and cell-type clustering validation with scRNA-seq data
Supplementary Figure 6. Additional measures of pooled AAV capsid and regulatory cargo profiles and summary
Supplementary Table 1. Gene list selected for comparison between USeqFISH and single-cell RNA sequencing
Supplementary Table 2. Probe sequences used for all experiments
Supplementary Table 3. Primer sequences used for titration of the miRNA TS pool

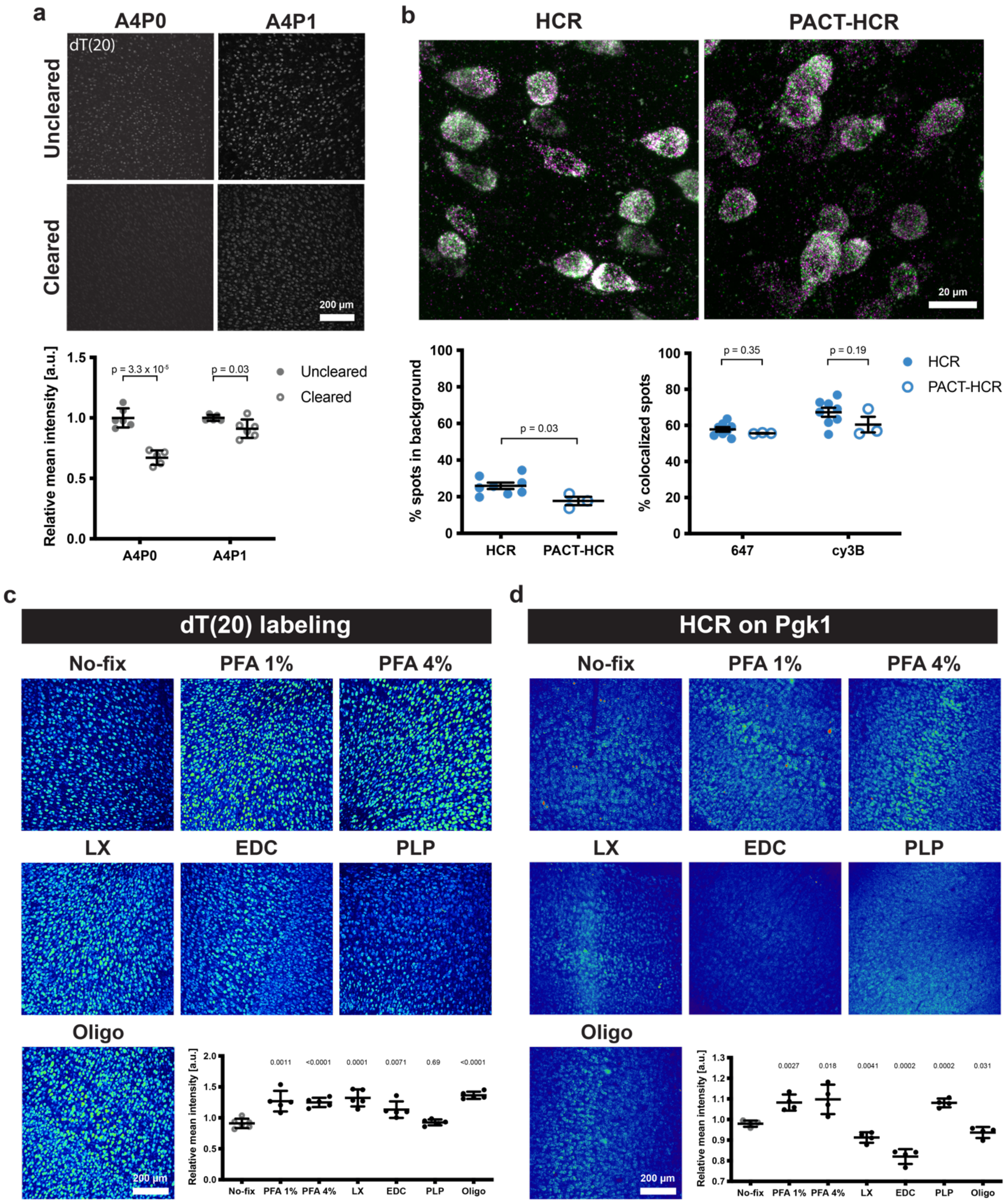

Supplementary Figure 1. Optimization of RNA-retained PACT. a, Comparison of PACT gels (A4P0: 4% acrylamide and no paraformaldehyde (PFA); A4P1: 4% acrylamide and 1% PFA) in retaining mRNA before ("Uncleared") and after detergent-based clearing (“Cleared”; two-sided unpaired t-test with Welch’s correction). The amount of total mRNA was estimated by labeling dT(20) conjugated with Alexa

Fluor 647. **b**, Comparison of detection efficiency of HCR applied to uncleared ("HCR") and PACT-cleared tissue ("PACT-HCR" with A4P1). We labeled *Gad1* using 12 probes (6 with cy3B (green) and the other 6 with Alexa Fluor 647 ("647", magenta)) to quantify the background and detection efficiency of the HCR and PACT-HCR, respectively (two-sided unpaired t-test with Welch's correction). **c**, Comparison of the total mRNA amount preserved in the PACT-cleared tissue (100 μm-thick) with A4P1 and additional fixatives (No-fix: no additional fixative used; LX: LabelX[41]; EDC: 1-Ethyl-3-3-dimentyl-aminopropyl carbodiimide[79]; PLP: periodate-lysine-PFA[80]; Oligo: Oligo(dT)$_{20}$-Acrydite). All additional fixation was performed by following the published protocol except Oligo; the samples were hybridized with oligo(dT)20-acrydite (IDT; 1 μM in 2xSSC) for 1 hr at room temperature before PACT clearing. **d**, Comparison of the HCR signal intensity labeling a house-keeping gene, *Pgk1*, in the PACT-cleared tissue with A4P1 and additional fixatives. Note that with A4P1, even though mRNA was better preserved with additional fixatives compared to No-Fix (**c**), some hindered probe hybridization (LX, EDC, and Oligo) or produced higher background (PLP) than others (**c, d**; unpaired t-t test compared to No-Fix).

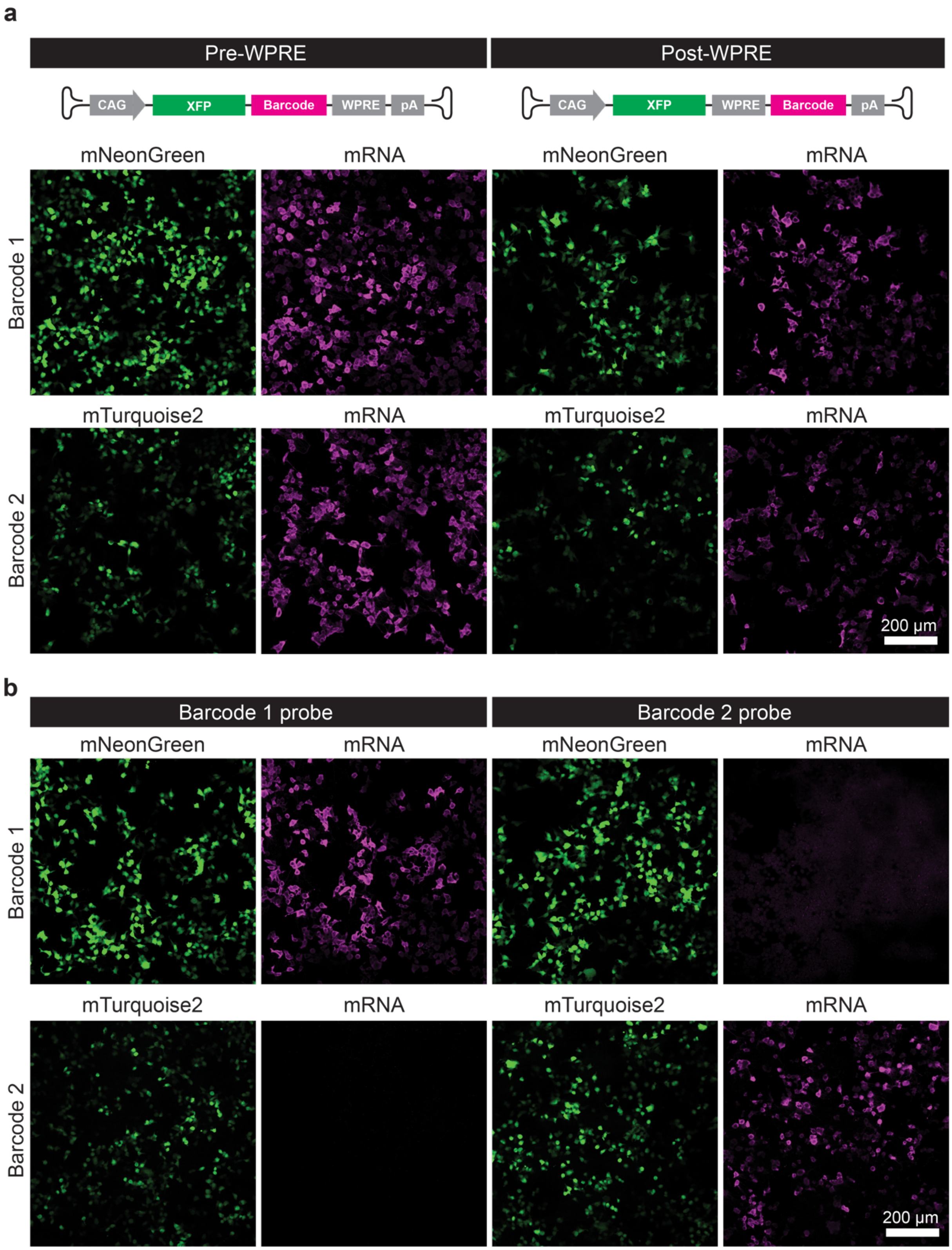

Supplementary Figure 2. Position and detection of barcodes inserted in the viral genome. a, Two barcodes (‘Barcode 1’ and ‘Barcode 2’) were inserted into two locations, between fluorescent protein

(XFP, either mNeonGreen or mTurquoise2) and WPRE (‘Pre-WPRE’) or between WPRE and pA (‘Post-WPRE’). After transfection into HEK293T cells, the barcodes were detected with the complementary probes. **b**, To examine the specificity of our barcode design, we tested if each probe for “Barcode 1” and “Barcode 2” could only hybridize to its corresponding barcode. In HEK293T cells transfected with each barcoded plasmid, we confirmed that the barcodes can only be detected by their corresponding probe, with negligible background.

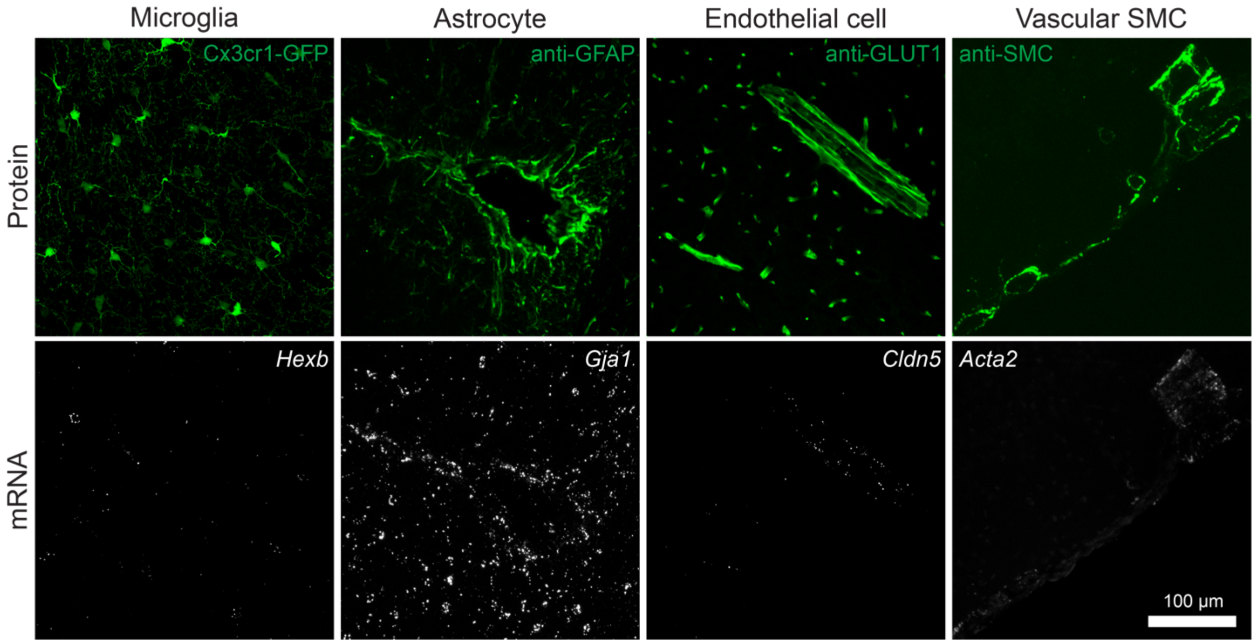

Supplementary Figure 3. Validation of probes for endogenous cell-type markers with post-hoc immunohistochemistry or transgenic reporter mouse lines. Probes designed to target some marker genes for non-neuronal cells were verified by observing co-localization of the RNA signal and protein either fluorescently tagged in transgenic mice (*Hexb* and Cx3cr1-GFP for microglia) or labeled with antibodies via immunohistochemistry (*Gja1* and anti-GFAP for astrocytes; *Cldn5* and anti-Glut1 for endothelial cells; *Acta2* and anti-SMC for vascular smooth muscle cells).

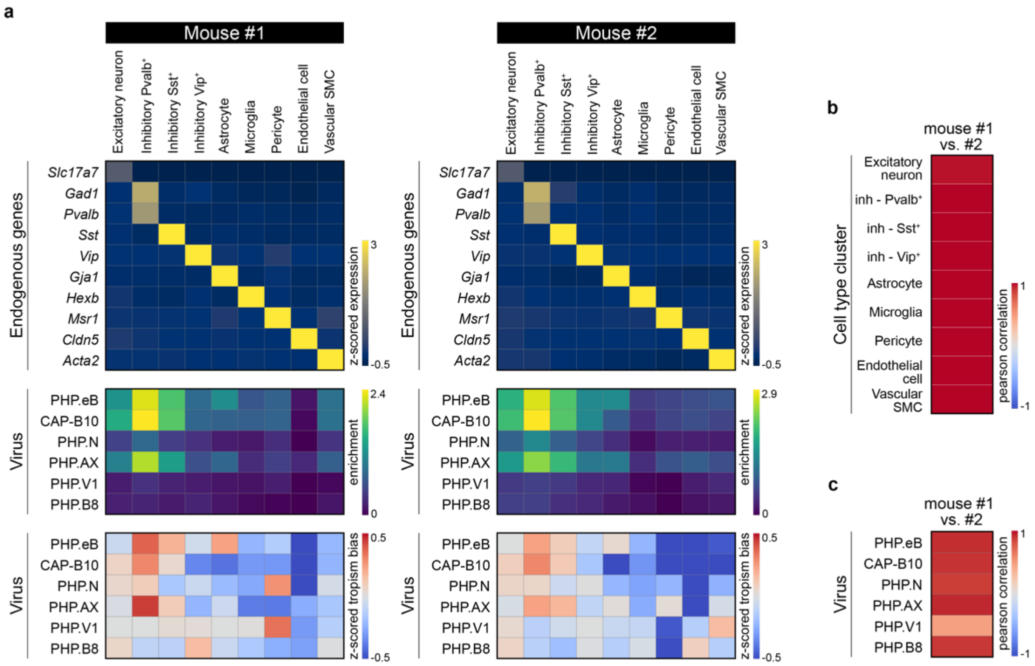

Supplementary Figure 4. Comparison of major cell type tropism profiling between two mice. To examine the reproducibility of USeqFISH-based AAV profiling, we separately analyzed the dataset used for Fig. 4 for each mouse. **a**. Endogenous (cividis) and viral gene expression (enrichment: viridis; relative tropism bias: coolwarm) profiles for each mouse. **b**, Pearson correlation of the mean endogenous gene expression levels for each cluster (the top heatmap in **a**) between two mice. **c**, Pearson correlation of the mean enrichment of AAVs for each (the middle heatmap in **a**) between two mice.

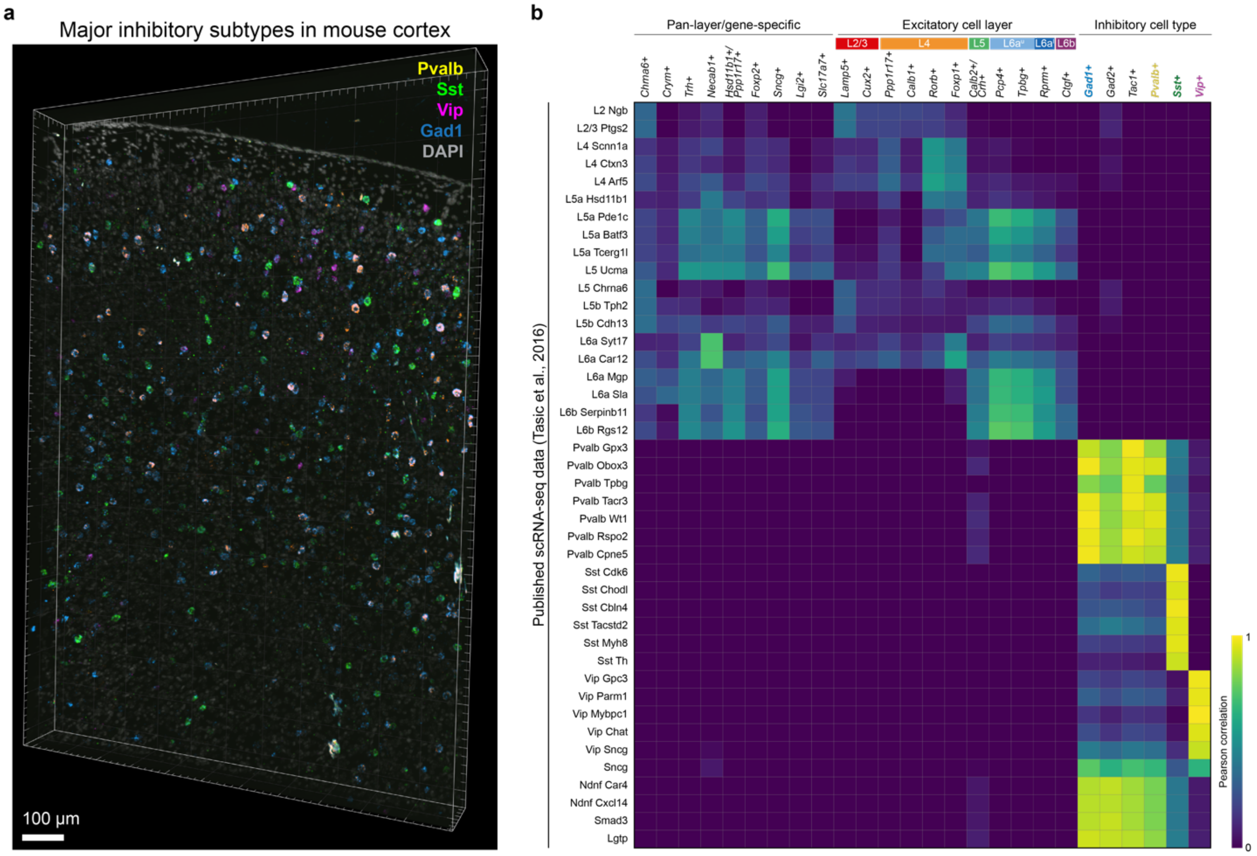

Supplementary Figure 5. USeqFISH detection of endogenous genes in mouse cortex volume and cell-type clustering validation with scRNA-seq data. a, 3D view of the cortex layer shown in Fig. 5a, highlighting that all the data were collected and analyzed in 3D. **b**, Pearson correlation of the mean gene expression levels for the clusters identified from the mouse cortical layer and neuronal clusters identified by publicly-available single-cell RNA sequencing data[63].

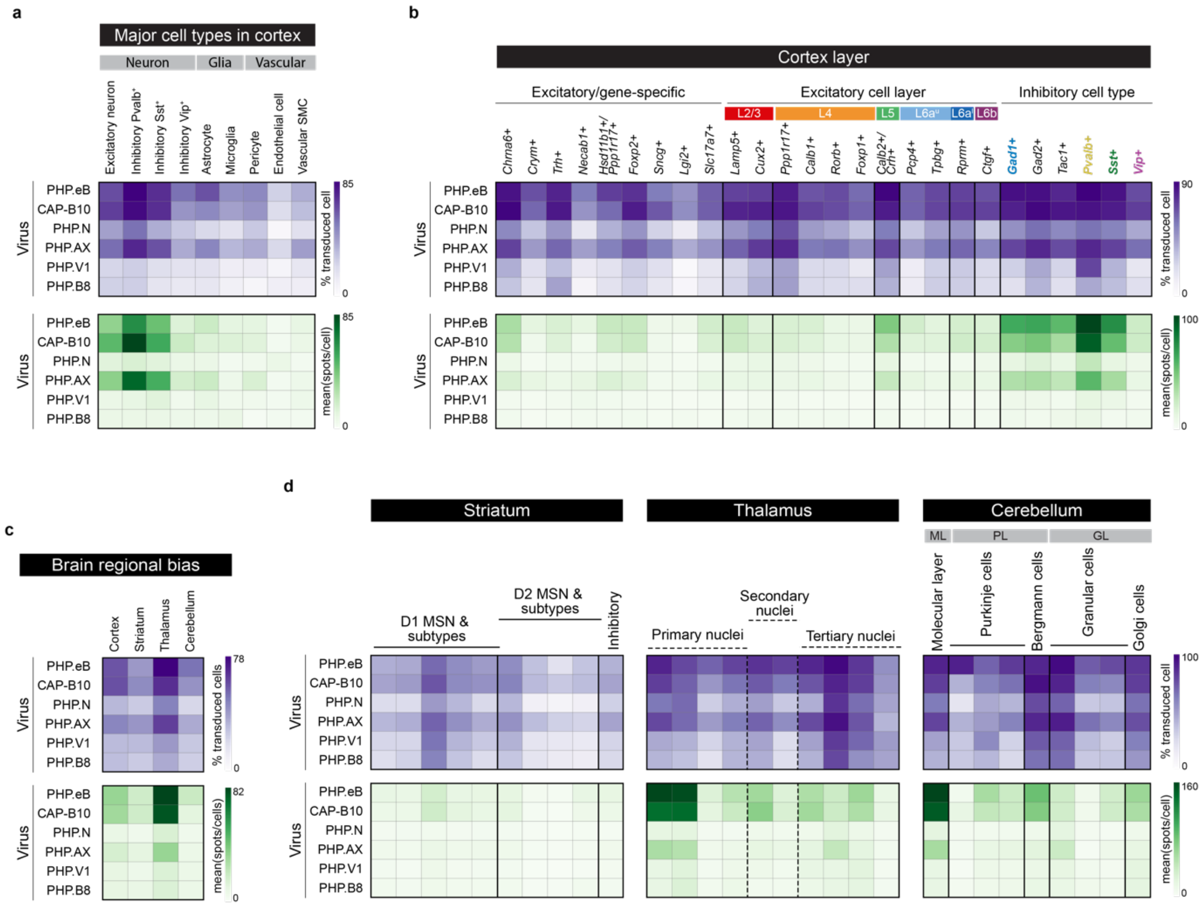

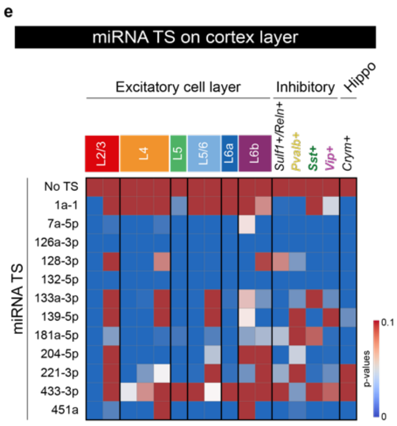

f

AAV	Transduction efficiency	Relative tropism bias (with CAG promoter)			
		Cortex	Striatum	Thalamus	Cerebellum
AAV-PHP.eB	++++	Inhibitory neurons, astrocytes	Broad coverage across D1 (Th^+) and D2 MSNs, inhibitory neurons	Primary nuclei (*Prkcd*$^+$)	ML, Purkinje cells
AAV.CAP-B10	++++	Excitatory and inhibitory neurons			ML, Bergmann cells, Golgi cells
AAV-PHP.N	++	Excitatory neurons		Tertiary nuclei (*Calb2*$^+$)	GL
AAV-PHP.AX	+++	Unbiased across neuronal subtypes + astrocyte			
AAV-PHP.V1	+	Vascular cells			
AAV-PHP.B8	+	-			

Supplementary Figure 6. Additional measures of pooled AAV capsid and regulatory cargo profiles and summary. a, b, c, d, Two additional profiling measurements for six systemically-delivered AAVs from all experiments: transduction rate (the number of cells expressing one viral spot or more over the total cell number in each cluster; purple), and transcription quantity (mean spot number per cell of only transduced cells; green) in **a**: major cortical cell types, related to Fig. 3d; **b**: cortical layer, related to Fig. 4b; **c**: brain regions, related to Fig. 4e; and **d**: cell-type clusters in the striatum, thalamus, and cerebellum,

related to Fig. 4f. **e**, P-values from the comparison of each miRNA TS to no TS control in each cell cluster (two-sided unpaired t-test) **f**, Summary of the transduction and relative tropism profiles of the six systemic AAVs investigated in this study with USeqFISH.
