# Supplementary material for: Spatial transcriptomics for profiling the tropism of viral vectors in tissues
Source: Nat Biotechnol. 2023 Jan 26;41(9):1272–86. doi: 10.1038/s41587-022-01648-w (PMC10443732; doi:10.1038/s41587-022-01648-w)
Supplement: Supplementary file 2 — Reporting Summary [file 41587_2022_1648_MOESM2_ESM.pdf]

nature research

Corresponding author(s): Viviana Gradinaru

Last updated by author(s): 12/08/2022

Reporting Summary

Nature Research wishes to improve the reproducibility of the work that we publish. This form provides structure for consistency and transparency in reporting. For further information on Nature Research policies, see our Editorial Policies and the Editorial Policy Checklist.

Statistics

For all statistical analyses, confirm that the following items are present in the figure legend, table legend, main text, or Methods section.

n/a	Confirmed	
☐	☒	The exact sample size (*n*) for each experimental group/condition, given as a discrete number and unit of measurement
☐	☒	A statement on whether measurements were taken from distinct samples or whether the same sample was measured repeatedly
☐	☒	The statistical test(s) used AND whether they are one- or two-sided<br>*Only common tests should be described solely by name; describe more complex techniques in the Methods section.*
☒	☐	A description of all covariates tested
☐	☒	A description of any assumptions or corrections, such as tests of normality and adjustment for multiple comparisons
☐	☒	A full description of the statistical parameters including central tendency (e.g. means) or other basic estimates (e.g. regression coefficient) AND variation (e.g. standard deviation) or associated estimates of uncertainty (e.g. confidence intervals)
☐	☒	For null hypothesis testing, the test statistic (e.g. F, t, r) with confidence intervals, effect sizes, degrees of freedom and P value noted<br>*Give P values as exact values whenever suitable.*
☒	☐	For Bayesian analysis, information on the choice of priors and Markov chain Monte Carlo settings
☒	☐	For hierarchical and complex designs, identification of the appropriate level for tests and full reporting of outcomes
☐	☒	Estimates of effect sizes (e.g. Cohen's *d*, Pearson's *r*), indicating how they were calculated

Our web collection on statistics for biologists contains articles on many of the points above.

Software and code

Policy information about availability of computer code

Data collection	For confocal imaging, we used Zeiss Zen or Andor Fusion 2.3. For sequential imaging, we used Andor Fusion 2.3.
Data analysis	For quantitative image analysis, we used custom python codes with Cellpose, SCANPY, Napari, and other python packages. All custom code and an example dataset to test are available at https://github.com/GradinaruLab/useqfish_probedesign (probe/barcode design for USeqFISH and HCR v3), https://github.com/GradinaruLab/useqfish_imaging (automated imaging and fluidics system control), and https://github.com/GradinaruLab/useqfish_analysis (image processing and data analysis). We used QX Manager software (Bio-Rad 12010213) for AAV titration.

For manuscripts utilizing custom algorithms or software that are central to the research but not yet described in published literature, software must be made available to editors and reviewers. We strongly encourage code deposition in a community repository (e.g. GitHub). See the Nature Research guidelines for submitting code & software for further information.

Data

Policy information about availability of data

All manuscripts must include a data availability statement. This statement should provide the following information, where applicable:

- Accession codes, unique identifiers, or web links for publicly available datasets
- A list of figures that have associated raw data
- A description of any restrictions on data availability

All sequences of probes and primers used in this study are provided in Supplementary Tables 2 and 3. We used Genbank genome assemblies (mm10 for mouse, cj1700 for marmoset, mmul10 for rhesus macaque) to build Bowtie2 databases for probe design. We used previously published data65 (Gene Expression Omnibus, GSE71585) in Supplementary Fig. 5b. The vector plasmid used to produce AAV-PHP.AX is available at Addgene (ID: 195218). Raw image datasets for pooled screening experiments are deposited to Brain Image Library (DOI). Other data that support the findings of this study are available from the corresponding author upon reasonable request.

Field-specific reporting

Please select the one below that is the best fit for your research. If you are not sure, read the appropriate sections before making your selection.

☒ Life sciences ☐ Behavioural & social sciences ☐ Ecological, evolutionary & environmental sciences

For a reference copy of the document with all sections, see nature.com/documents/nr-reporting-summary-flat.pdf

Life sciences study design

All studies must disclose on these points even when the disclosure is negative.

Sample size	No sample size calculation was performed. The animal numbers were determined by availability. The number of cells used in this study were determined by automated segmentation of collected images.
Data exclusions	No data were excluded from the analyses.
Replication	All in vitro experiments were repeated at least three times with similar results. All in vivo experiments with mice were repeated more than twice using 2-5 animals with similar results. Supplementary Fig 4 supports the reproducibility of our results across animals. All NHP experiments were repeated at least twice using 1 animal with similar results. We confirmed the reproducibility of our method by applying the same code and analysis to the dataset of each animal and showed high correlation between the results.
Randomization	All mice were randomly selected to be administered with viruses. All non-human primates were selected according to their availability at experimental time points. For imaging we randomly selected the fields-of-views in the specific region of the brain we selected.
Blinding	Blinding was unnecessary for USeqFISH development. For pooled AAV profiling, unique gene identifies were assigned to each variant or gene for probe design, used for experimental design and process, and matched after data analysis. For all experiments, no subjective measurement on data collection was involved.

Reporting for specific materials, systems and methods

We require information from authors about some types of materials, experimental systems and methods used in many studies. Here, indicate whether each material, system or method listed is relevant to your study. If you are not sure if a list item applies to your research, read the appropriate section before selecting a response.

Materials & experimental systems

n/a	Involved in the study	
☐	☒	Antibodies
☐	☒	Eukaryotic cell lines
☒	☐	Palaeontology and archaeology
☐	☒	Animals and other organisms
☒	☐	Human research participants
☒	☐	Clinical data
☒	☐	Dual use research of concern

Methods

n/a	Involved in the study	
☒	☐	ChIP-seq
☒	☐	Flow cytometry
☒	☐	MRI-based neuroimaging

Antibodies

Antibodies used	Aves GFP-1020, 1:1000<br>anti-GFAP, 829401, BioLegend, 1:1000<br>anti-Glut1, 07-1401, Millipore Sigma, 1:400<br>anti-actin, alpha-smooth muscle-cy3, C6198, Millipore Sigma 1:1000<br>anti-Pvalb, ab181086, Abcam, 1:200<br>goat anti-chicken IgY, Alexa Fluor 633, A21103, Invitrogen, 1:1000<br>goat anti-rabbit IgG, Alexa Fluor 633, A21070, Invitrogen, 1:1000<br>donkey anti-rabbit IgG antibody conjugated with Alexa Fluor 647, 711-605-152, Jackson ImmunoResearch, 1:200
Validation	All antibodies were independently validated and confirmed to be co-localized with respective RNA signals in Supplementary Fig. 3 and Extended Data Fig. 9b. We also verified that they have no signal in the absence of the primary antibody.

Eukaryotic cell lines

Policy information about cell lines

Cell line source(s)	HEK293T cells (ATCC, CRL-3216)

Cell line source(s)	NIH3T3 cells (ATCC, CRL-1658)
Authentication	None of the cell lines used were authenticated.
Mycoplasma contamination	The cell lines were not tested for mycoplasma contamination.
Commonly misidentified lines (See ICLAC register)	No cell lines used in this studies are commonly misidentified.

Animals and other organisms

Policy information about studies involving animals; ARRIVE guidelines recommended for reporting animal research

Laboratory animals	We purchased C75BL/6j (000664) mice (all males, 6-8 weeks old) from Jackson Laboratory. Mice were locally housed in the animal facility at Caltech and managed at 71-75 F, the humidity of 30% - 70% with the lighting cycle of 13 hours on & 11 hours off. The detailed information of animal cares and procedures for the marmoset and the rhesus macaques are available in references 29 and 86 (Chuapoco et al., Biorxiv, 2022; Chen et al., Neuron, 2022)
Wild animals	The study did not involve wild animals.
Field-collected samples	The study did not involve field-collected samples.
Ethics oversight	For mice, all animal procedures were approved by the California Institute of Technology Institutional Animal Care and Use Committee (IACUC). For marmosets, all animal procedures were approved by the University of California, San Diego, IACUC and in accordance with National Institutes of Health and the American Veterinary Medical Association guidelines. For rhesus macaques, all animal procedures were approved by the Institutional Animal Care and Use Committee at the University of California, Davis and the California National Primate Research Center (CNPRC)

Note that full information on the approval of the study protocol must also be provided in the manuscript.
